# Genomic Action of Sigma-1 Receptor Chaperone Relates to Neuropathic Pain

**DOI:** 10.1007/s12035-020-02276-8

**Published:** 2021-01-18

**Authors:** Shao-Ming Wang, Nino Goguadze, Yuriko Kimura, Yuko Yasui, Bin Pan, Tzu-Yun Wang, Yoki Nakamura, Yu-Ting Lin, Quinn H. Hogan, Katherine L. Wilson, Tsung-Ping Su, Hsiang-en Wu

**Affiliations:** 1 Cellular Pathobiology Section, Integrative Neuroscience Research Branch, Intramural Research Program, National Institute on Drug Abuse, NIH/DHHS, Suite 3512, 333 Cassell Drive, Baltimore, MD 21224 USA; 2 Department of Anesthesiology, Medical College of Wisconsin, Milwaukee, WI 53226 USA; 3 Department of Psychiatry, College of Medicine, National Cheng Kung University, Tainan City, 70101 Taiwan; 4 Department of Pharmacology, Graduate School of Biomedical & Health Science, Hiroshima University, Hiroshima, 734-8553 Japan; 5 Department of Cell Biology, Johns Hopkins University School of Medicine, Baltimore, MD 21205 USA

**Keywords:** Sigma-1 receptor, Neuropathic pain, Dorsal root ganglia, Spare nerve injury, Translational inhibition, 4E-BP1

## Abstract

Sigma-1 receptors (Sig-1Rs) are endoplasmic reticulum (ER) chaperones implicated in neuropathic pain. Here we examine if the Sig-1R may relate to neuropathic pain at the level of dorsal root ganglia (DRG). We focus on the neuronal excitability of DRG in a “spare nerve injury” (SNI) model of neuropathic pain in rats and find that Sig-1Rs likely contribute to the genesis of DRG neuronal excitability by decreasing the protein level of voltage-gated Cav2.2 as a translational inhibitor of mRNA. Specifically, during SNI, Sig-1Rs translocate from ER to the nuclear envelope via a trafficking protein Sec61β. At the nucleus, the Sig-1R interacts with cFos and binds to the promoter of 4E-BP1, leading to an upregulation of 4E-BP1 that binds and prevents eIF4E from initiating the mRNA translation for Cav2.2. Interestingly, in Sig-1R knockout HEK cells, Cav2.2 is upregulated. In accordance with those findings, we find that intra-DRG injection of Sig-1R agonist (+)pentazocine increases frequency of action potentials via regulation of voltage-gated Ca2+ channels. Conversely, intra-DRG injection of Sig-1R antagonist BD1047 attenuates neuropathic pain. Hence, we discover that the Sig-1R chaperone causes neuropathic pain indirectly as a translational inhibitor.

**Supplementary Information:**

The online version contains supplementary material available at 10.1007/s12035-020-02276-8.

## Introduction

Neuropathic pain is one of the most debilitating forms of chronic pain due to its lancinating nature with unpredictable and spontaneous episodes [1]. Current treatments for neuropathic and chronic pain are unsatisfactory [2, 3]. There is a consensus that pathological changes in primary sensory neurons cause pain due to increased excitability [4–6]. Neuronal hyperexcitability and its underlying mechanism(s) in primary sensory neurons are intensively studied to address the opioid overdose epidemic that afflicts this country like a plague. Peripheral sensory neurons are readily available to a variety of drug delivery modes, including direct delivery into hyperactive dorsal root ganglia (DRG). This treatment route is highly effective, efficient, and well tolerated as compared to systemic administration [7], identifying primary sensory neurons as key targets to treat neuropathic pain [8].

The sigma-1 receptor (Sig-1R) is a ligand-mediated multifunctional chaperone protein that typically resides at the mitochondria-associated ER membrane (MAM), and is enriched in DRGs [9, 10]. Sig-1Rs are ligand- and stress-driven, and can translocate from the MAM to other subcellular locations to modulate diverse pathways and proteins [9, 11, 12] [13] [14, 15] including ion channels [16] and receptors on the plasma membrane [17, 18].

Sig-1R has a critical role in neuropathic pain [19]; nerve injury–induced hyperalgesia is prevented by intrathecal blockade of Sig-1R, and in mice lacking Sig-1R [20, 21]. Further, Sig-1R activating neurosteroids can influence pain at the spinal level [22, 23]. However, the exact molecular mechanism relating Sig-1R to the genesis or processing of neuropathic pain is largely unknown. Here, we focused on DRGs and determined if the Sig-1R affects neuronal hyperexcitability in those neurons and, if so, how.

Dysfunction of voltage-gated calcium channels (VGCCs) in the primary sensory neurons is known to contribute to neuropathic pain [24] [5, 25]. Previous studies showed that Sig-1R regulates the activity of VGCCs [26–28]. In this report, we examined the molecular mechanism whereby Sig-1Rs might negatively regulate VGCCs.

We used the spare nerve injury (SNI) model to generate neuropathic pain in rats and found that the Sig-1R regulates Cav2.2 (N-type of VGCCs) by reducing the translation of Cav2.2 mRNA by an upstream mechanism that controls the availability of a global 5′-cap-binding factor, eukaryotic initiation factor 4E (eIF4E), required for translation [29]. Our findings link Sig-1R to the genesis of neuropathic pain, which implicate Sig-1R as a feasible therapeutic target for neuropathic pain. Further, we found that the intra-DRG injection of Sig-1R antagonist attenuates SNI-induced neuropathic pain and propose here that this procedure is a feasible route in clinical setting to treat neuropathic pain.

Those results are presented in this report.

## Methods

### Animals

All methods and animal procedures were conducted in accordance with the principles as indicated by the *NIH Guide for the Care and Use of Laboratory Animals*. These animal protocols were also reviewed and approved by the NIDA intramural research program Animal Care and Use Committee, National Institute of Health, and Medical College of Wisconsin Institutional Animal Care and Use Committee. Male Sprague-Dawley rats (Charles Rivers or Taconic Farms Inc.) were housed in a room maintained at 22 ± 0.5 °C and constant humidity (60 ± 15%) with an alternating 12-h light-dark cycle. Food and water were available ad libitum throughout the experiments.

### Sensory Testing

To examine the behavioral responses after intraganglionic injection in rats, four sensory tests were measured at different time points after the injection in the sequence described previously [30]. Firstly, brush (2.5-mm width, Ted Pella Inc., Redding, CA) was applied by light brushing along the bottom of the right hind paw from front to rear for 3 times with 5-s intervals in between. Secondly, the cold stimulation was initiated with the application of a drop of acetone touching the plantar surface using a PE20 attached syringe for 3 times, with 2 min in between. For both brush and cold stimulation, a positive response was recorded if any paw withdrawal occurred. Thirdly, mechanical withdrawal threshold was determined using von Frey filaments of eight different forces (0.40, 1.19, 2.05, 3.63, 5.5, 8.65, 15.0, and 29.0 g; Smith and Nephew Inc., Germantown, WI). The up-and-down method was used to determine the 50% withdrawal threshold [31]. Finally, the noxious stimulation was applied using a 22G spinal needle with adequate force to indent but not penetrate the skin for 10 stimuli, applied at intervals of 10 s. For each application, the induced behavior was either a very brisk, simple withdrawal with immediate return of the foot to the cage floor, or a sustained elevation with grooming that included licking and chewing, and possible shaking, which lasted at least 1 s [32]. To examine the behavioral changes after nerve injury (SNI), only noxious stimulation with pin was measured before (baseline) and on the day of experiment (tissue collection). After nerve injury, only rats that displayed a hyperalgesia-type response of at least 2 out of the 10 stimuli were used further in this study.

### Intraganglionic Microinjection

We have previously reported that direct microinjection into the DRG for delivering a compound is a reliable method to affect sensory function at a segmental level [30]. Briefly, after paravertebral exposure and minimal foraminotomy, 2 μl of agent was injected into L4 and L5 DRG through a pulled small-tip glass micropipette attached to a microprocessor-controlled injector (Nanoliter 2000, World Precision Instruments, Sarasota, Florida) over 5 min.

### Nerve Injury Models

Rats weighing 125 to 150 g were subjected to either SNI or spinal nerve ligation (SNL) under anesthesia with 2% isoflurane in oxygen. (1) In the SNI model, the mid-thigh sciatic nerve was exposed, and the tibial and common peroneal branches were individually ligated with 6.0 silk suture and cut distally to the ligature. The sural nerve was preserved [33]. (2) In the SNL model [34], the right paravertebral region was exposed. The L6 transverse process was removed, after which the L5 and L6 spinal nerves were ligated with 6-0 silk suture and transected distal to the ligature. In both models, the muscular fascia was closed with 4-0 resorbable polyglactin sutures and the skin was closed with staples. Control animals received skin incision and closure only. The SNL model was used in the measurement of [Ca^2+^]_c_ experiment. Otherwise, the SNI model was employed.

### Intact Ganglion Tissue Preparation

The preparation of intact ganglion with attached dorsal root for electrophysiological study was described previously [35]. Briefly, under isoflurane anesthesia, a laminectomy was executed while bathing the surgical area with oxygenated artificial cerebrospinal fluid (aCSF (in mM); NaCl, 128; KCl, 3.5; MgCl_2_, 1.2; CaCl_2_, 2.3; NaH_2_PO_4_, 1.2; NaHCO_3_, 24.0; glucose, 11.0; pH 7.35 with CO_2_). The L4 or L5 ganglion with attached dorsal root was isolated from naïve rats. After removing the surrounding DRG capsule, the tissue preparation was transferred to the recording chamber circulated with aCSF at 37 °C.

### DRG Neuron Dissociation and Plating

For Ca^2+^ microfluorometry and action potential (AP) generation, DRGs were rapidly harvested from ipsilateral L4 and L5 DRGs during isoflurane anesthesia with decapitation at 21 to 28 days after SNL or from sham surgery animals [32]. DRGs were incubated in 0.5 mg/ml Liberase TM (Roche, Indianapolis, IN) in DMEM/F12 with glutaMAX for 30 min at 37 °C, followed with 1 mg/ml trypsin and 150 Kunitz units/ml DNase (Sigma-Aldrich) for another 10 min. After addition of 0.1% trypsin inhibitor, tissues were centrifuged, lightly triturated in neural basal media (1 ×) (Life Technologies) containing 2% (v:v) B27 supplement (50 ×) (Life Technologies), 0.5 mM glutamine, 0.05 mg/ml gentamicin, and 10 ng/ml nerve growth factor 7S (Alomone Labs Ltd., Jerusalem, Israel). Cells were then plated onto poly-l-lysine-coated glass cover slips and incubated at 37 °C in humidified 95% air and 5% CO_2_ for at least 2 h and were studied 3–6 h after dissociation.

### Electrophysiological Recording

To measure AP properties, intracellular recordings from intact ganglion tissue preparations were harvested as described above. Transmembrane potentials were recorded in sensory neuron somata with a pulled glass electrode (70–100 MΩ) containing 2 M potassium acetate. Traces were filtered at 10 kHz and digitized at 40 kHz (Digidata 1332A, Axon Instruments, San Jose, CA), using an active bridge amplifier (Axoclamp 2B; Axon Instruments) and an upright microscope. To evoke APs, a square-wave pulse (1-ms duration) with 1.5 times the intensity of threshold was applied to the dorsal root. Afterhyperpolarization (AHP) was measured from the resting membrane potential to the most hyperpolarized level of the AHP. The AHP duration is defined as 50% of time required to return from the AHP maximum to the resting membrane potential.

For the AP generation experiment, whole-cell current clamp recordings of dissociated neurons were made using a patch clamp amplifier (MultiClamp 700B; Molecular Devices, Sunnyvale, CA). Patch pipettes, ranging from 2 to 4 MΩ resistance, were formed from borosilicate glass and filled with the internal pipette solution containing (in mM) 130 K-Glucolate, 5 KCl, 2 MgCl_2_, 0.2 EGTA, 10 HEPES, 4 Mg-ATP, and 0.3 Na_2_-GTP, at pH of 7.2 with KOH and osmolarity of 296 to 300 mOsm. Soma of the dissociated sensory neurons was depolarized directly by current injection via the recording electrode. Signals were filtered at 2 kHz and sampled at 10 kHz with a Digidata 1440A digitizer and pClamp10 software (Molecular Devices). Series resistance (5–10 MΩ) was monitored before and after the recordings, and data were discarded if the resistance changed by 20%.

### Measurement of [Ca^2+^]_c_

The [Ca^2+^]_c_ was measured according to our previous publication [36]. Briefly, neuron-plated cover slips were exposed to Fura-2-AM (5 μM) for 30 min at room temperature, washed 3 times with regular Tyrode’s solution, and given 30 min for de-esterification. For Ca^2+^ microfluorometry, the fluorophore was excited alternately with 340-nm and 380-nm wavelength illumination (150W Xenon, Lambda DG-4, Sutter, Novato, CA), and images were acquired at 510 nm using a cooled 12-bit digital camera (Coolsnap fx, Photometrics, Tucson, AZ) and inverted microscope (Diaphot 200, Nikon Instruments, Melville, NY). Recordings from each neuron were obtained as separate regions (MetaFluor, Molecular Devices, Downingtown, PA) at a rate of 3 Hz. After background subtraction, the fluorescence ratio R for individual neurons was determined as the intensity of emission during 340-nm excitation (I_340_) divided by I_380_, on a pixel-by-pixel basis. The [Ca^2+^]_c_ was then estimated by the formula K_d_·β·(*R*–*R*_min_)/(*R*_max_−*R*) where β = (I_380max_)/(I_380min_). Traces were analyzed using Axograph X 1.1 (Axograph Scientific, Sydney, Australia). Activation-induced transients were generated by depolarization produced by microperfusion application of KCl 50 mM for 3 s.

### Cell Culture and Transfection

Human embryonic kidney (HEK293T) cells and mouse neuroblastoma Neuro-2a (N2a) cells were obtained from ATCC and were cultured according to the method described previously [37]. Briefly, both HEK and N2a cells were maintained in Dulbecco’s modified Eagle’s medium (GIBCO) supplemented with penicillin (100 units/mL), streptomycin (100 μg/mL), and 10 % Fetalgro bovine growth serum (RMBIO). The maintained culture medium was also supplemented with puromycin (100 μg/ml) for the Sig-1R knockout (KO) HEK cells [38]. PolyJet (SignaGen Lab) was used in transfection according to the manufacture’s manual. Plasmid vectors used were pCMV6-CACNA1B-myc/DDK (Origene Technologies Inc., Rockville, MD; Cat. RC217170), pCMV6-EIF4EBP1-myc/DDK (Origene Technologies Inc., Rockville, MD; Cat. RC201348), pCMV6-FOS-myc/DDK (Origene Technologies Inc., Rockville, MD; Cat. RC202597), pCMV6-myc/DDK (Origene Technologies Inc., Rockville, MD; Cat. PS100001), pCMV6-MZF1-myc/DDK (Origene Technologies Inc., Rockville, MD; Cat. RC220791), pCMV6-OCT1-myc/DDK (Origene Technologies Inc., Rockville, MD; Cat. RC208599), pCMV3-GFP control vector (Sino Biological, Wayne, PA; Cat. CV026), pCMV3-HA-Sec61β□ (Sino Biological, Wayne, PA; Cat. MG51753-NY), pCMV3-HA-SP3 (Sino Biological, Wayne, PA; Cat. HG13964-CY), pCMV3-Sigma-1R-GFP (Sino Biological, Wayne, PA; Cat. MG57873-ACG), pCMV3-HA control vector (Sino Biological, Wayne, PA; Cat. CV017), pcDNA3-HA-CEBPD (self-construct), pcDNA3-HA-SOX2 (self-construct), and Sigma-1R-EYFP (self-construct).

### Sig-1R Knockout HEK Cells

Wild-type (WT) and Sig-1R KO HEK cells were generated by using the CRISPR KO system [38]. Briefly, Human Sig-1R CRISPR/Cas9 KO and Sig-1R HDR plasmids (Santa Cruz) were co-transfected in HEK293T cells using PolyJet transfection reagent (SignaGen). To select Sig-1R CRISPR/Cas9-KO HEK cells, cells were maintained in puromycin (100 μg/ml, GIBCO) containing cell culture medium as described above to generate permanent Sig-1R-KO HEK cells.

### Western Blot Analysis

Total protein was extracted from L4, L5, and L6 DRGs from skin sham and SNI animals or cultured cells. Harvested ganglia were homogenized with 200 μl RIPA lysis buffer (50 mM Tris, pH7.4; 150 mM NaCl; 0.2% sodium deoxycholate; 0.1% SDS; 1% Triton X-100) containing protease inhibitors (Roche Diagnostics, Indianapolis, IN), and protein amount was measured (Micro BCA Protein Assay Kit, ThermoScientific). Equal amount (30 μg) of proteins were denatured with SDS 4 × sample buffer (Bio-Rad) at a final volume of 40 μl or 50 μl containing 1% 2-mercaptoethanol and heated at 95 °C for 10 min or 37 °C for 15 min. These protein samples were separated by using SDS-polyacrylamide gel electrophoresis (SDS-PAGE) and transferred onto a polyvinylidene difluoride membrane. After incubation with 5% nonfat milk in TBST (Tris-buffered saline with 0.1% Tween 20) for 1 h, membranes were incubated with various primary antibodies overnight at 4 °C, which were used as loading control. Membranes were washed with TBST 3 times for 15 min followed by probing with secondary antibody. Blots were washed 3 times for 15 min with TBST and developed by using the LiCor system (LiCor Odyssey CLx) or the Azure Biosystem C600 and band intensity was analyzed by Image Studio Lite (LiCor 5.2.5) according to the manufacturer’s manual. Primary and secondary antibodies used were as follows: anti-Sig-1R antibody (rabbit; 1:1000 dilution; Lot No. 5460, custom-made polyclonal antibody raised against peptide143-165 from the N-terminus of rat Sig-1R), anti-Sig-1R (B5) mouse monoclonal antibody (1:1000 dilution; Santa Cruz Biotechnology, SC-137075), anti-Cav1.2 antibody (rabbit; 1:200 dilution; Alomone Lab, ACC-003), CACNA1B rabbit polyclonal antibody (1:200 dilution; Proteintech Inc., 19681-1-AP), anti-Na^+^-K^+^ ATPase ( mouse, 1:500 dilution; Santa Cruz, SC-21712), anti-GAPDH (rabbit monoclonal antibody, 1:1000 dilution; Cell Signaling, 5174S), anti-eIF4E (rabbit polyclonal antibody, 1:1000 dilution; MBL, RN001P), anti-4E-BP1 (1: 1000 dilution; Cell Signaling, 9644S), anti HDAC2 antibody (mouse monoclonal antibody, 1:1000 dilution, Cell Signaling, 5113S), anti-GFP (1:1000 dilution; Proteintech, 66002-1), anti-HA (1:5000 dilution; Proteintech, 51064-2-AP), anti-Sec61β (1:500 dilution; Invitrogen, PA3-015), anti-multi ubiquitin antibody (mouse monoclonal antibody, 1:1000 dilution, Stressgen, SPA-025), anti-DDK (mouse monoclonal antibody, 1:2000 dilution, Origene, TA50011-100), anti-Myc-tag (mouse monoclonal antibody, 1:2000 dilution; Cell Signaling, 2276S), anti-Myc-tag (rabbit monoclonal antibody, 1:2000 dilution; Cell Signaling, 2278S), anti-c-FOS (rabbit monoclonal antibody, 1:1000 dilution; Cell Signaling, 2250S), and anti-N-Cadherin (1:500 dilution; Santa Cruz, SC-7939) overnight at 4 °C. α-Tubulin (1: 20000 dilution, Sigma-Aldrich, T5168) was used as loading control. Membranes were washed with TBST 3 times for 15 min followed by probing with secondary antibody of goat anti-rabbit (1:15000 dilution, LiCor, IRDye 800CW), goat anti-mouse antibody (1:15000 dilution, LiCor, IRDye 680RD), peroxidase-conjugated AffiniPure goat anti-mouse IgG (1:10000 dilution; Fc fragment specific; Jackson ImmunoResearch Lab, 111-035-164), peroxidase-conjugated AffiniPure goat anti-mouse IgG (1:10000 dilution; light chain specific; Jackson ImmunoResearch Lab, 111-035-174), or peroxidase-conjugated AffiniPure goat anti-rabbit IgG (1:10000 dilution; Jackson ImmunoResearch Lab, 111-035-046).

### Biotinylation of Cell Surface Protein

The L4, L5, and L6 DRGs from skin sham and SNI animals were harvested at 14 days after surgery. After removing the surrounding capsule/tissue, DRGs were chopped with small scissors followed by being washed once with ice-cold PBS (pH 8.0; with protease inhibitor). The samples were incubated with biotinylation reagent (2 mg; EZ-Link Sulfo-NHS-Biotin; ThermoScientific) in 1 ml cold PBS (pH 8.0) rotated at 4 °C for 3 h. After washing twice with cold TBS (pH 7.4), the sample was homogenized with immunoprecipitation (IP) lysis buffer (50 mM Tris, pH 7.4; 150 mM NaCl; 1% Nonidet P40) and then sonicated at 90% strength for 10 s (total 8 times for control and 6 times for SNI) followed by incubation at 4 °C for 30 min. After centrifugation, samples were measured for protein content. The biotinylated lysates received streptavidin beads (200 μl; streptavidin magnetic beads, New England Biolab) and were incubated at 4 °C overnight with rotation. After washing with IP lysis buffer (10 min for 3 times; 50 mM Tris, pH8.0, 120 mM NaCl, 0.5% Nonidet P40), samples were eluted with 60 μl 2 × sample buffer (Bio-Rad) containing 1% 2-ME by heating at 60 °C for 20 min. Proteins were separated by running SDS/PAGE gels as described above.

### Immunoprecipitation

Lysate from DRG tissue: The L4, L5, and L6 DRGs from skin sham and SNI animals were homogenized with 300 μl IP lysis buffer containing protease inhibitors (Roche Diagnostics, Indianapolis, IN). Lysate from cell lines: HEK-293T cells were harvested in 0.3 ml of IP lysis buffer for 30 min. Protein amounts were measured (Pierce Bicinchoninic Acid Protein Assay Kit, ThermoFisher Scientific, Rockford, IL) after centrifugation (14,000 rpm for 10 min at 4 °C). Protein lysate (150 or 200 μg) was precleared by incubating with 50 μl protein-A/G agarose beads (Santa Cruz Biotechnology) for 1 h at 4 °C with rotation. The mixture was centrifuged at 10,000 rpm for 1 min. Specific antibody (2 μg; see the list of antibodies) based on experiments or control IgG was added into the precleared lysates and rotated for 2 h at 4 °C. Subsequently, the lysate containing antibody was supplemented with protein-A/G agarose beads (50 μl) to a total amount of 1000 μl and rotated overnight at 4 °C. The beads were washed three times with IP lysis buffer that contained protease inhibitor for 5 min at 4 °C and each wash was followed by centrifugation at 10,000 rpm for 1 min at 4 °C to remove the supernatant. After the 3rd wash, the bound proteins were eluted with 50 μl SDS 2 × sample buffer (Bio-Rad) containing 1% 2-ME and heated at 95 °C for 10 min. The resulting proteins were immediately resolved by using SDS/PAGE and immunoblotted with primary antibody overnight at 4 °C. Membranes were washed 3 times (15 min each) followed by probing with specific secondary antibody as described above. Blots were washed 3 times (15 min each) with TBST and developed using the Azure Biosystem C600. Band intensity was analyzed by Image Studio Lite (LiCor 5.2) according to the manufacturer’s manual.

### RNA Immunoprecipitation

The RNA immunoprecipitation was performed according to the manufacture’s manual (Imprint® RNA Immunoprecipitation Kit; Sigma-Aldrich, St. Louis, MO). Briefly, control or Sig-1R KO HEK cells were harvested in lysis buffer containing protease inhibitor cocktail and ribonuclease inhibitor, and incubated on ice for 15 min. After centrifugation, the lysate was incubated with eIF4E antibody (15 μg; MBL, RN001P) or normal IgG antibody (15 μg; Cell Signaling) overnight at 4 °C. Samples were then incubated with magnetic beads (DynaBeads Protein A, Invitrogen by ThermoFisher Scientific, Waltham, MA) at 4 °C for 2 h for immunoprecipitation. Samples were washed 5 times with washing buffer. The RNA was then purified by miRCURY^TM^ RNA isolation kit (EXIQON, product #300110; Woburn, MA). A reverse transcription reaction (RNA to cDNA EcoDry^TM^ Premix kit; Clontech Laboratories, Cat#639543; Mountain View, CA) was performed to synthesize the complementary DNA and quantitative PCR was conducted using Syber Green Master Mix (Roche) and specific primers to quantify the cDNA level.

### Chromatin Immunoprecipitation

Chromatin immunoprecipitation (IP) assay was conducted based on the method described previously [39]. Briefly, the Sig-1R-GFP or cFOS-Myc/DDK expressing HEK cells were fixed with 1% formaldehyde for 20 min. The cross-linked chromatin of the collected cells was sonicated to generate chromatin fragments between 300 and 1000 bp. The fixed fragmented DNA proteins were immunoprecipitated with anti-GFP, Myc, or negative control IgG at 4 °C for 18 h. After reversal of the crosslinking between proteins and genomic DNA, precipitated and purified DNA was amplified by PCR. The primer sequences for human 4E-BP1 promoter in the PCR were as follows: (F): 5′-GGTCAAGAAATTGAAGCGGG-3′; (R) 5′-GATGGCGGGCGGGATAGCTC-3′.

### Quantitative Real-Time PCR

Total RNA was obtained from L4-L6 DRGs of control and SNI animals (3, 14, and 28 days after surgery) or from wild-type or KO Sig-1R HEK293T cells following the manufacturer’s instruction (RNeasy mini kit; Qiagen). cDNA was synthesized from total RNA using RNA to cDNA EcoDry mastermix (Takara Bio USA, Inc.). Quantitative real-time PCR was carried out by using SYBR Green Master Mix (Roche) with specific primers to quantify the cDNA level of Sig-1R, Cav1.2, Cav2.2, and 4E-BP1. Glyceraldehyde 3-phosphate dehydrogenase (GAPDH) [40] served as the reference gene for normalization. For each sample, triplicate determinations were averaged and the fold differences in SNI expression were compared to those of sham surgery samples using the comparative C_T_ method. ΔΔC_T_ values were used for statistical analysis, while 2^−ΔΔCT^ values were used to plot the graph for Sig-1R, Cav1.2, Cav2.2, and 4E-BP1 gene expression in different groups. Primers used are listed below. (1) Rat CACNA1B; (F) GCGAGAACTGAATGGGTACTT, (R) GGACTTCTCTTCTGCGTTCTT (NM_001195199.1; IDT, Coralville, IA); (2) Human CACNA1B; (F) GAGTGGCCTCCATTCGAGTA, (R) CCCAGAGCGATGATTTTGAT (NM_147141.1; IDT); (3) Rat CACNA1C; (F) ACAAGTGGGATAGCTGTTCAGT, (R) CCTCAGACAGGCAACTGGAG (NM_012517.2; IDT); (4) Human 4E-BP1; (F) ATTTAAAGCACCAGCCATCG, (R) TGGAGGCACAAGGAGGTATC (NM_004095.4; IDT); (5) Rat 4E-BP1; (F) CACAGCAGTCAGGCCTTGTA, (R) CAGGGAGGGTGTAGGTGAGA (NM_053857.2; IDT); (6) Human GAPDH; (F) GAGTCAACGGATTTGGTCGT, (R) GACAAGCTTCCCGTTCTCAG (NM_001357943.2; IDT); (7) Rat GAPDH; (F) ATGACTCTACCCACGGCAAG, (R) CATACTCTGCACCAGCATCTC (NM_017008.4; IDT); (8) Rat Sigma-1R; (F) TACCATCATCTCTGGCACTTTC, (R) AACCGTCTCTCCTGGGTAATA ( NM_030996.1; IDT).

### Immunohistochemical Experiment on DRGs

The rat DRGs were fixed and harvested according to previous publication [41] with modification. Briefly, after anesthetized with isoflurane (Isothesia; Henry Schein) and trans-cardiac perfused with 0.1 M phosphate buffer (PB: pH 7.4) followed by 4% paraformaldehyde (w/v; Sigma-Aldrich) in 0.1 M PB, L4, L5, and L6 DRGs were harvested and post-fixed in formaldehyde solution rotated overnight at 4 °C and sent for paraffin embedding and sectioned (5 μm; AML Laboratories, Jacksonville, FL). Tissue sections were deparaffinized, rehydrated, and then processed for antigen retrieval as follows. The slides were deparaffinized sequentially using xylene solutions and rehydration with serial dilutions of ethanol (100 to 70%). After washing out ethanol with tap water, slides underwent antigen retrieval as follows: after pre-incubation with 0.01 M citrate buffer pH 6.0 for 10 min at room temperature, the slides were heated in the pre-warmed buffer at 95 °C, followed by post-incubation for about 20 min to bring the solution to room temperature. The antigen retrieval was performed in 50 mL polypropylene conical tube (Falcon, ref 352098). After washing with TBS, sections were blocked with 5% normal goat serum (NGS, Invitrogen, Cat. 31872) in TBS containing 0.1% Tween 20 (v/v) for 1 h at room temperature. The sections were then incubated with the mouse anti-Sig-1R (B5, mouse monoclonal antibody, 1:200-1:500, Santa Cruz, SC137075), rabbit anti-Sec61β (1: 250, ThermoScientific, PA3-015), and Lamin A/C rabbit antibody (1:100, Cell Signaling, Cat. 2032S) in the blocking solution overnight at 4 °C. Following three 10-min TBS washes, sections were incubated with Alexa Fluor 488-conjugated goat anti-rabbit IgG or Alexa Fluor 564-conjugated goat anti-mouse IgG (1:500, Invitrogen, Cat. A11029) in 5% NGS in TBS for 90 min at room temperature in the dark. The sections were washed with PBS for 5 min three times, then counterstained with 4′,6-diamino-2-phenylindole (DAPI, Invitrogen, 1 μg/mL) by 10-min incubation at room temperature. Sections were washed with PBS for 5 min three times, mounted with Prolong Diamond Antifade Mountant (Life technologies, Carlsbad, CA) and coverslips. PerkinElmer confocal microscope Modular Laser system 2.0 with Nikon Eclipse TE2000E microscope and Volocity version 6.3 for data acquisition, or Zeiss confocal microscope LSM 710 system with Zeiss AX10 microscope and Zen version 2.3 SP1 for data acquisition, was used to examine both the immunohistochemical and immunocytochemical experiment. The Photoshop (Adobe Photoshop CC 2019, version 20.0.10) and Image J software were used for image processing subsequent to data acquisition.

### Immunocytochemical Experiment on Cell Lines

In N2a cells, Sig-1R-EYFP and/or HA-Sec61β expressing N2a cells were fixed with 4% paraformaldehyde (Sigma-Aldrich) in PBS as described previously [37] for 20 min. After washing with PBS 5 min three times, sections were blocked with 5% NGS in PBS containing 0.1% Tween 20 (v/v) for 1 h at room temperature. After washing with PBS for 5 min three times, the fixed cells were immunostained with mouse anti-Sec61β (1:200, Santa Cruz, SC393633), or mouse anti-emerin (1:200, Santa Cruz, Cat. SC81552) at 4 °C overnight. After washing, cells were incubated with the 2nd antibody (1:300, AlexaFluor 488- or Alexa Fluor 568-conjugated antibody, Invitrogen, Cat. A11029, A11008, A11030, A11037). After 3 times of 5-min PBS washing, sections were mounted with Prolong Gold Antifade Mountant (Life technologies, Carlsbad, CA). PerkinElmer confocal microscope Modular Laser system 2.0 with Nikon Eclipse TE2000E microscope and Volocity version 6.3 for data acquisition were used to examine the immunocytochemical results.

### Statistical Analysis

Prism (version 8.2, GraphPad Software, Inc., San Diego, CA) was used to perform Student’s *t* test, paired *t* test, Mann-Whitney test, Fisher’s exact test, or two-way ANOVA. Main effects identified by ANOVA were further analyzed by Tukey’s post hoc or Sidak’s multiple comparisons tests. Unless specified, data were derived from at least three animals’ DRGs or three independent experiments from cultured cell line for every group. Data are reported as mean ± SEM. A *P* value of less than 0.05 was considered significant. Traces from Ca^2+^ microfluorometry and intracellular recordings were analyzed by using Axograph X 1.4.4 (Axograph Scientific).

## Results

### Cav2.2 Protein Level is Decreased After SNI

**Fig. 1:**
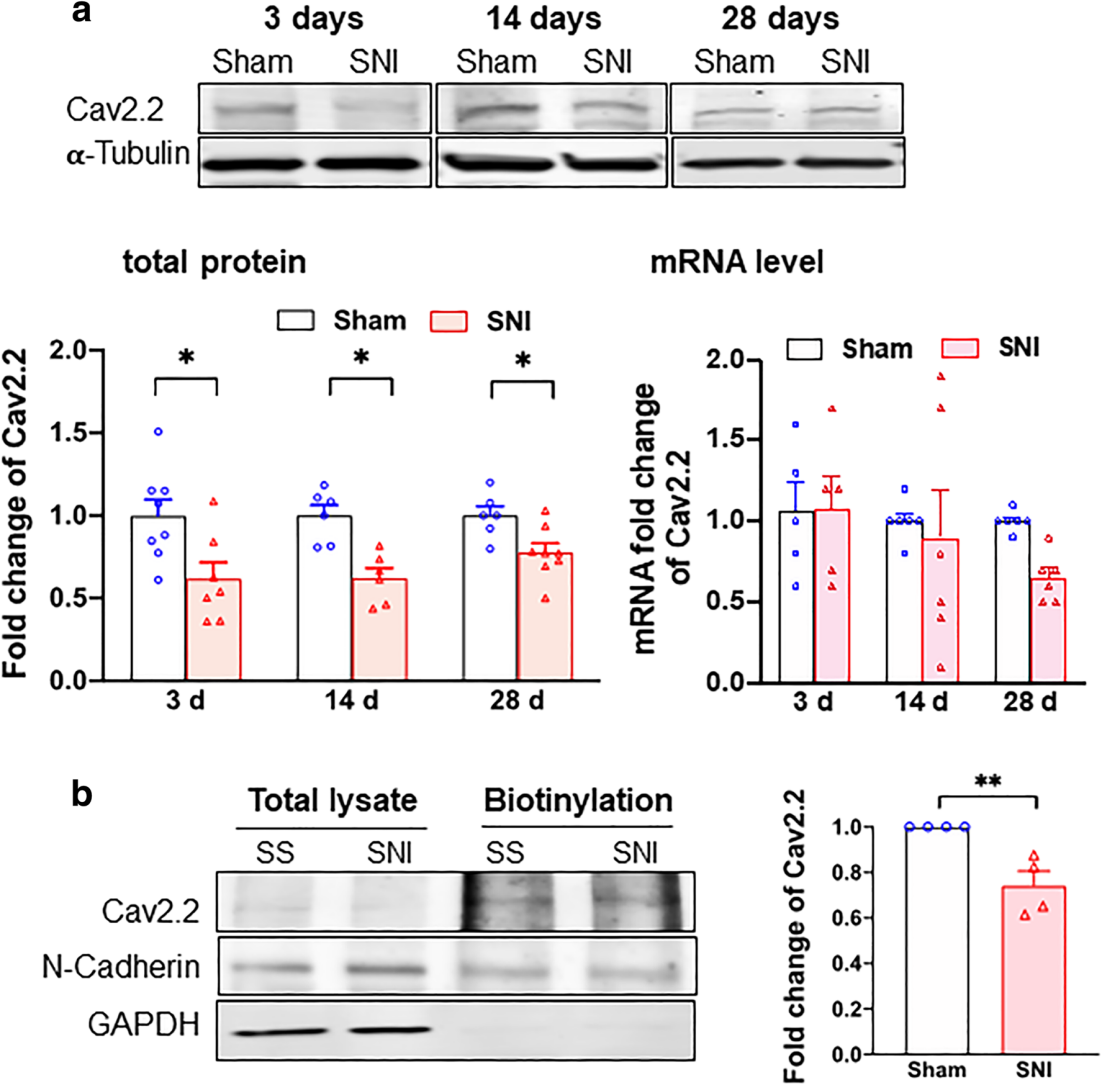
Cav2.2 protein expression is downregulated in DRGs after SNI. **a** Total Cav2.2 protein expression deceases after SNI. Sample bands of Cav2.2 expression were demonstrated at different time after sham surgery or SNI from rat DRGs. α-Tubulin served as internal control. Summary data showed that Cav2.2 protein expression but not mRNA level decreases after SNI on day 3, 14, or 28. **b** Cav2.2 protein level decreases at the plasma membrane after SNI. Cav2.2 at plasma membrane decreases after day 14 of SNI. N-Cadherin expression from streptavidin beads pulled down served as internal control. Data are means ± SEM; two-way ANOVA, or Student’s *t* test; *P < 0.05, **P < 0.01

It is known that the SNI-induced DRG neuronal hyperexcitation relates to VGCCs. We therefore examined VGCCs in sham vs SNI DRGs. There was no alteration at either the protein or mRNA level of Cav1.2 at 3, 14, and 28 days after the SNI (Supplementary Figure S1A and S1B). The protein level of Cav2.2 after SNI, however, is significantly reduced (Fig. 1a). Notably, the Cav2.2 mRNA level is not significantly altered as judged by ANOVA although there is a tendency of reduction on day 28 after SNI (Fig. 1a). Those results suggest a reduced translation of Cav2.2 mRNA after the SNI. Importantly, the level of Cav2.2 on cellular surface in DRGs apparently decreases after SNI as seen in the day 14 sample per determination by the biotinylation assay (Fig. 1b).

**Fig. 2:**
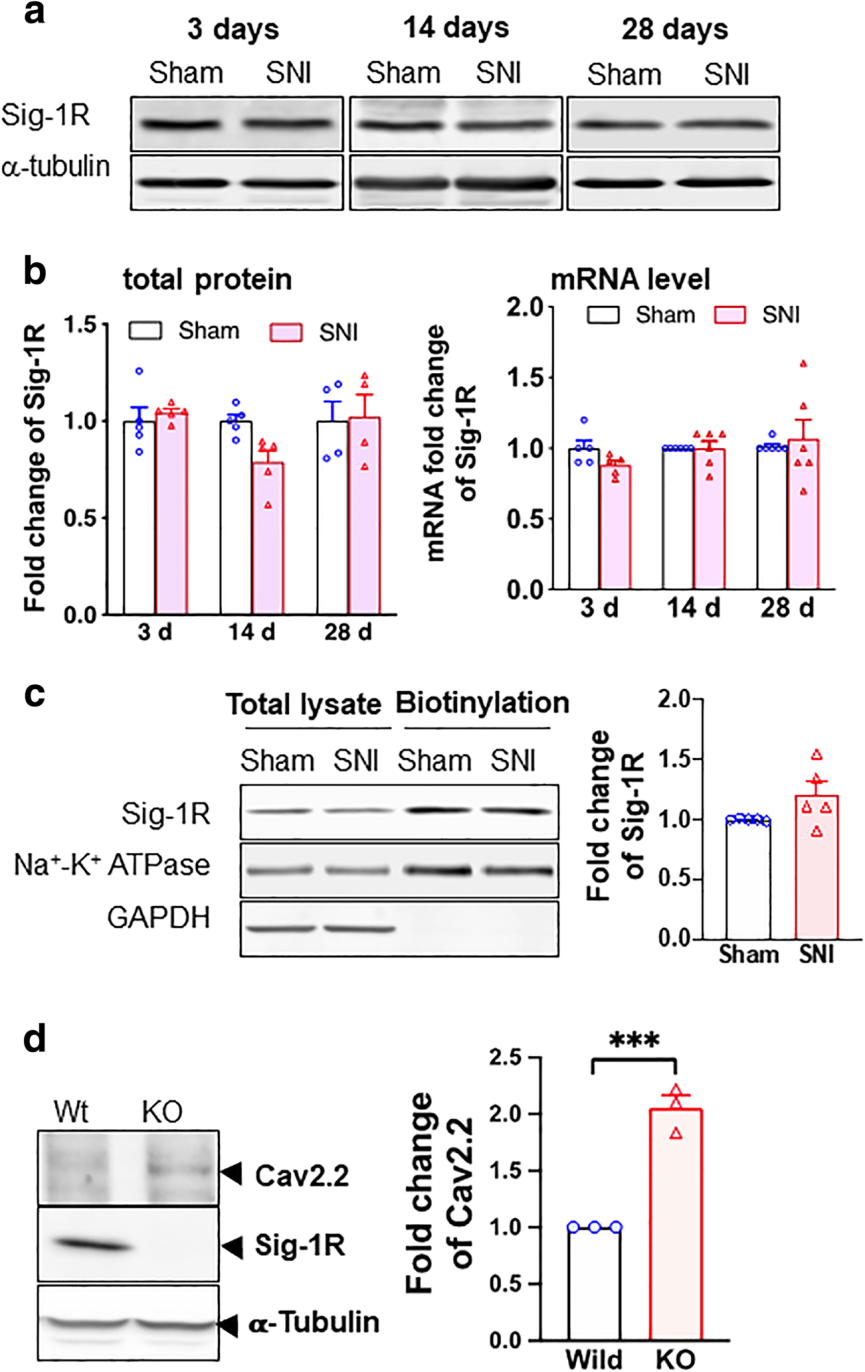
Sigma-1 receptor transcription, protein expression, and surface level do not change after SNI in rat DRGs. **a** Sample blots of sigma-1 receptor (Sig-1R) expression at different time points after SNI. α-Tubulin served as internal control. **b** Summary data show that Sig-1R protein expression decreases slightly at day 14 (nonsignificant per ANOVA) but not at days 3 or 28 after SNI (left panel). The Sig-1R mRNA does not change after SNI at days 3, 14, or 28 (right panel). **c** Surface level of Sig-1Rs remains unchanged after SNI. Streptavidin beads were used to pull down biotinylated lysate (200 μg) from DRGs 14 days after SNI or sham surgery. Na^+^-K^+^ ATPase served as internal control and GAPDH served as negative control. **d** Sig-1R KO increases Cav2.2 expression in HEK cells. Western blotting shows an increase of Cav2.2 protein level in total cellular lysates of Sig-1R KO HEK cells when compared to wild-type cells. Data represent means ± SEM; two-way ANOVA or Student’s *t* test

We examined also the expression of Sig-1Rs after SNI. The protein level of Sig-1R stays the same after SNI at all time points (Fig. 2a) and the level of its mRNA is likewise unchanged (Fig. 2b). Further, the cellular surface level of Sig-1R of DRGs does not differ between SNI and sham DRGs (Fig. 2c).

Is the Sig-1R related to the level of Cav2.2 anyway? By comparing the level of Cav2.2 in wild-type vs Sig-1R knockout (Sig-1R KO) HEK cells, we found that indeed the Sig-1R affects the level of Cav2.2. Cav2.2 level is increased in Sig-1R KO cells (Fig. 2d). Thus, the Sig-1R certainly plays a negative role in the expression of Cav2.2.

We hypothesized that the Sig-1R might influence the translation of Cav2.2 mRNA as a potential mechanism to reduce Cav2.2 protein levels, for example as seen in SNI.

We examined next if the Sig-1R, typically an ER-localized chaperone at the ER-mitochondrion interface called the MAM, may regulate the translation of the Cav2.2 mRNA and if so through what mechanism.

### Sec61β May Assist in Moving Sig-1Rs from ER to the Nucleus

Sig-1Rs can translocate from the MAM to nuclear envelope to recruit chromatin remodeling molecules to regulate gene transcription [37]. However, the potential mechanism underlying the translocation of Sig-1Rs from ER to nuclear envelope remains unknown. Sec61β is a protein transport protein that plays a role in the receptor translocation from the plasma membrane or ER to the nuclear envelope [42, 43]. We asked therefore if Sig-1Rs translocate to nuclear envelope through a similar route.

**Fig. 3:**
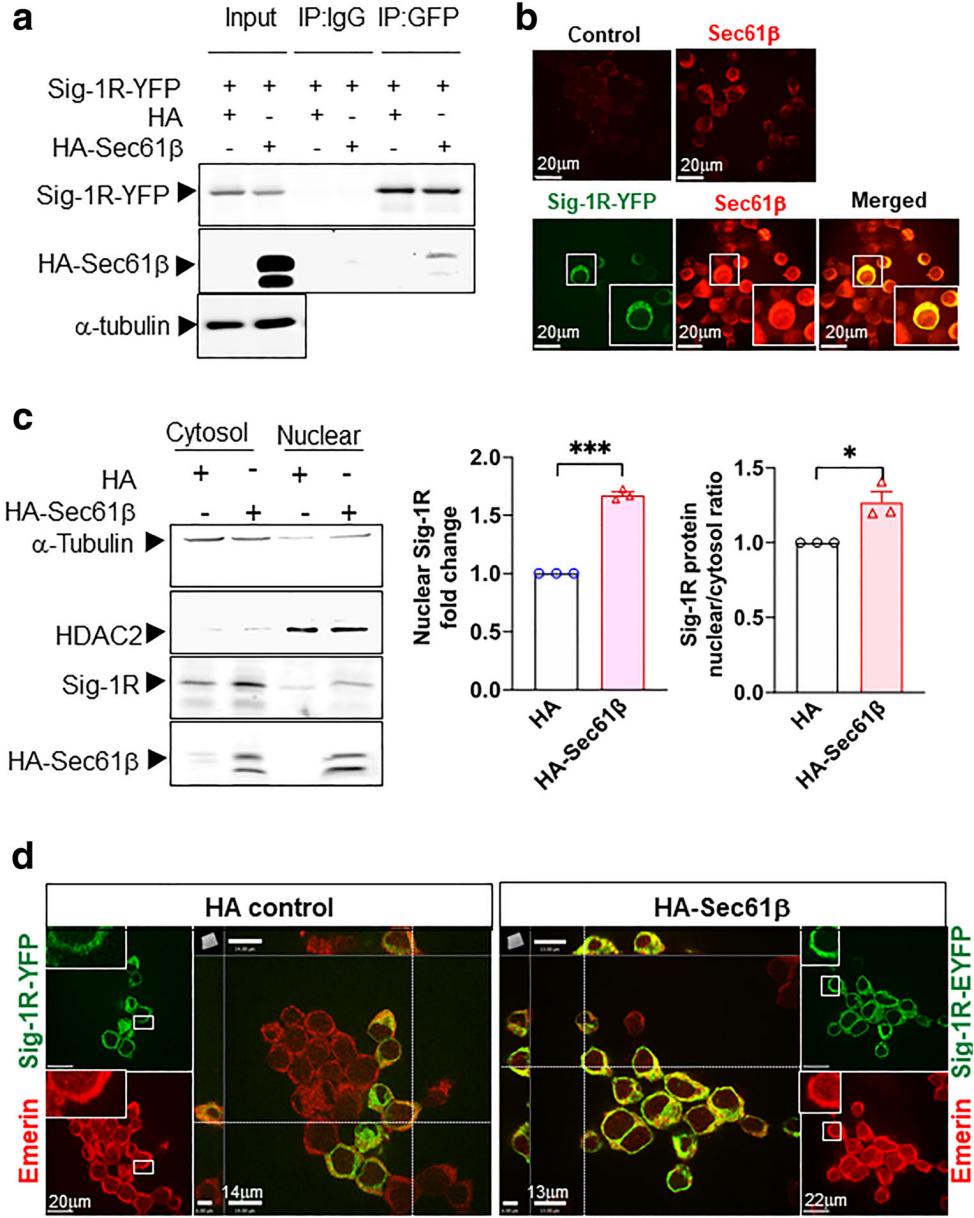
Sig-1R translocates to the nuclear envelope via Sec61β. **a** Sig-1R interacts with Sec61β. In N2a cells, HA-tagged Sec61β and Sig-1R-EYFP were overexpressed. Immunoprecipitation with GFP antibody shows an interaction between Sig-1R and Sec61β. **b** Sig-1R colocalizes with endogenous Sec61β. Confocal images demonstrate colocalization of immunoreactive EYFP-tagged Sig-1R (green) and Sec61β (red) in Sig-1R-EYFP overexpressing N2a cells. **c** Increase of Sig-1R in nuclear fraction in HA-Sec61β-expressing N2a cells. Subcellular fractionation followed by western blotting shows an increase of Sig-1R in the nuclear fraction (left and middle panels) as reflected in the nuclear/cytosol ratio (right panel). **d** Colocalization of Sig-1R and emerin in Sig-1R-EYFP and HA-Sec61β overexpressing N2a cells. Confocal images indicate perinuclear colocalization of immunoreactive EYFP-tagged Sig-1R (green) and emerin (red). Data are shown as means ± SEM; Student’s *t* test; *P < 0.05, ***P < 0.001

**Fig. 4:**
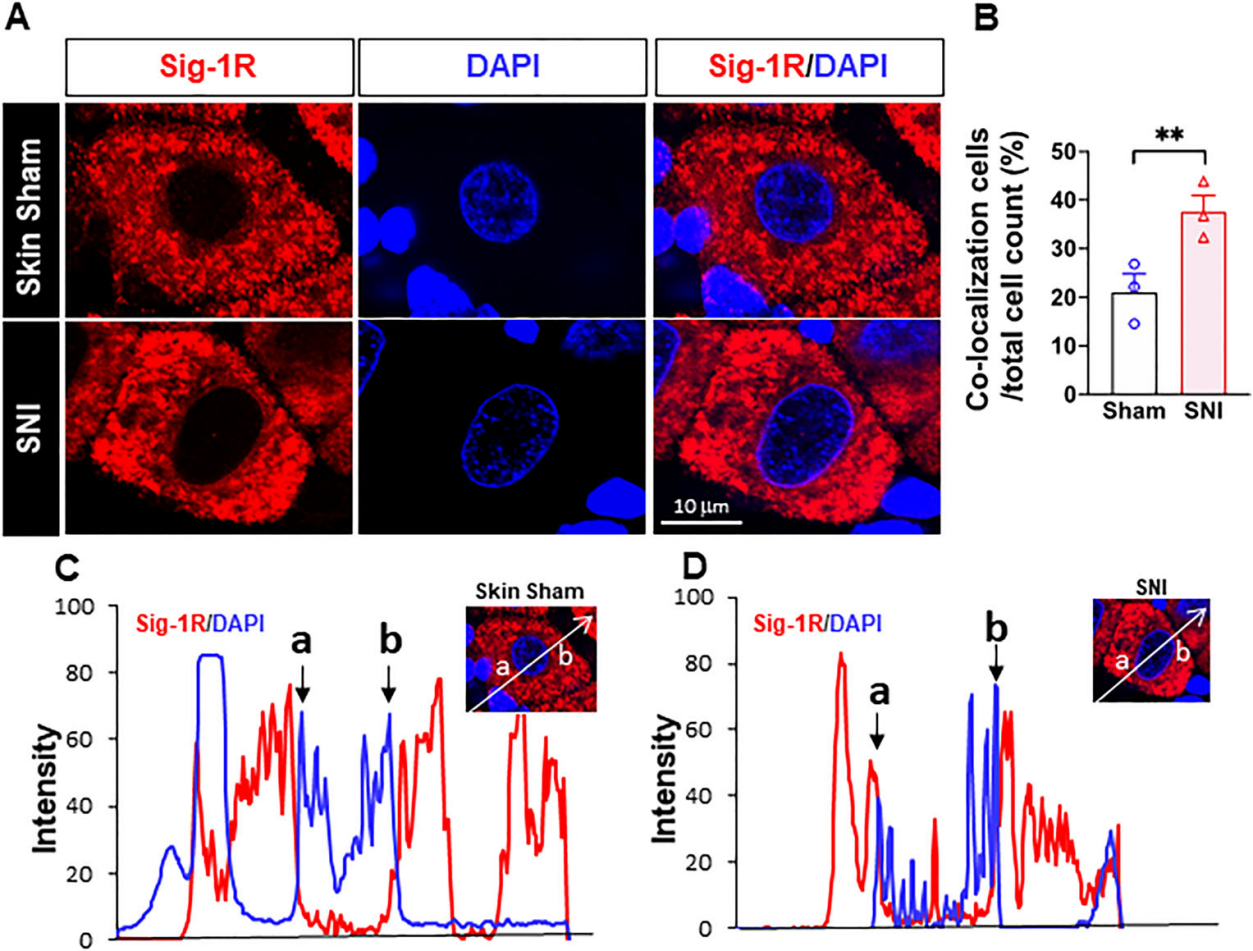
More Sig-1Rs are seen at the nuclear envelope after SNI. (a) Confocal images show clear localization of Sig-1R (red) near DNA maker 4’-6-diamidino-2-phenylindole (DAPI; blue) in respective places in the sham or SNI rat DRGs. (b) Summary data on neurons which demonstrate colocalization of Sig-1R and DAPI in skin sham or SNI DRG. (c, d) Traces of Sig-1R (red) and DAPI (blue) in the two cells shown in (A) simply to show that the cell in (D) showed a presumed nucleopasmic reticulum-associated ‘sipke’ of Sig-1R after SNI. Data are shown as means ± SEM. The means were calculated from the ratio of “Sig-1R and DAPI colocalized neurons” to total of neurons counted in each animal; number in bars represents animal number. Note: in the 3 sham animals, 37 of 172 neurons show observed colocalization; in SNI animals, 55 of 151 neurons show as such. Two experimenters made the decision, on single blind basis, on the colocalization of Sig-1R and DAPI by eyeballing images like those in (A). The image provider then calculated the results obtained from the two persons. Fisher’s exact test was used to test the difference; **P < 0.05

We found that HA-Sec61β interacts and colocalizes with Sig-1R in Sig-1R-EYFP overexpressing N2a cells (Fig. 3a, b). Note that endogenous Sec61β interacts with Sig-1R-GFP as well (Supplementary Figure S2). To confirm that Sec61β modulates the Sig-1R translocation, we examined the subcellular distribution pattern of both molecules. Results show that in HA-Sec61β-overexpressing N2a cells, the endogenous Sig-1R increases, unexpectedly through an unknown mechanism, in total lysate (Supplementary Figure S3). Nevertheless, the nuclear to cytosol ratio of Sig-1R increases in HA-Sec61β-overexpressing cells when compared to controls receiving only the HA vector (Fig. 3c). In Sec61β-overexpressing N2a cells, a majority of Sig-1R-YFP colocalizes with Emerin, a nuclear inner membrane protein [44] (Fig. 3d). The quantification of Sig-1R-GFP colocalization with emerin is shown in the supplementary figure (Figure S4). These data suggest that Sig-1Rs translocate from ER to the nuclear inner membrane per Sec61β. Note that our finding here is consistent with Sig-1R localization at the nucleoplasmic reticulum as we confirmed [45] in the next figure (Fig. 4).

### Sig-1R in Close Proximity to the Nuclear Envelope of DRG After SNI

Sig-1Rs signals are higher near the nuclear envelope based on staining with 4′,6-diamidino-2-pheynlindole (DAPI) in DRG on day 14 after SNI, compared to that seen in skin-sham DRG (Fig. 4(A, B)). Our results are also consistent with the report that Sig-1Rs are enriched at the nucleus after SNI in mice [46], and that Sig-1Rs localize in infolded regions of the nuclear envelope (“nucleoplasmic reticulum”; [45]). We also observed an increase of the colocalization of Sig-1R with Sec61β in SNI DRG when compared to the sham DRG (Supplementary Figure S5).

Note the distinct existence of Sig-1Rs in the nucleoplasmic reticulum in this study (the red spike between a and b in Fig. 4(D)). In this study, we used B5 monoclonal antibody with the paraffin embedding procedure that has been reported [41] to be an essential step to maintain the specificity of the B5 antibody as illustrated in a comparative staining.

### Cav2.2 mRNA Translation is 5′Cap-Dependent and Involves 4E-BP1 and eIF4E

Although no single mechanism controls the translation of all mRNAs, one particular mechanism affecting the initiation of mRNA translation is the so-called cap-dependent initiation in which binding of translation initiation factors (eIFs; eIF4E for example) to the cap of the 5′ end of mRNA plays a critical role in the initiation of translation [29]. There are eIF-binding proteins, 4E-BP1 for example, that can bind eIF and lock it up to prevent an initiation of mRNA translation [47]. 4E-BP1 has been shown to relate to neuropathic pain at the DRGs (review see [48]) and is upregulated at the injured sciatic nerve [49].

**Fig. 5:**
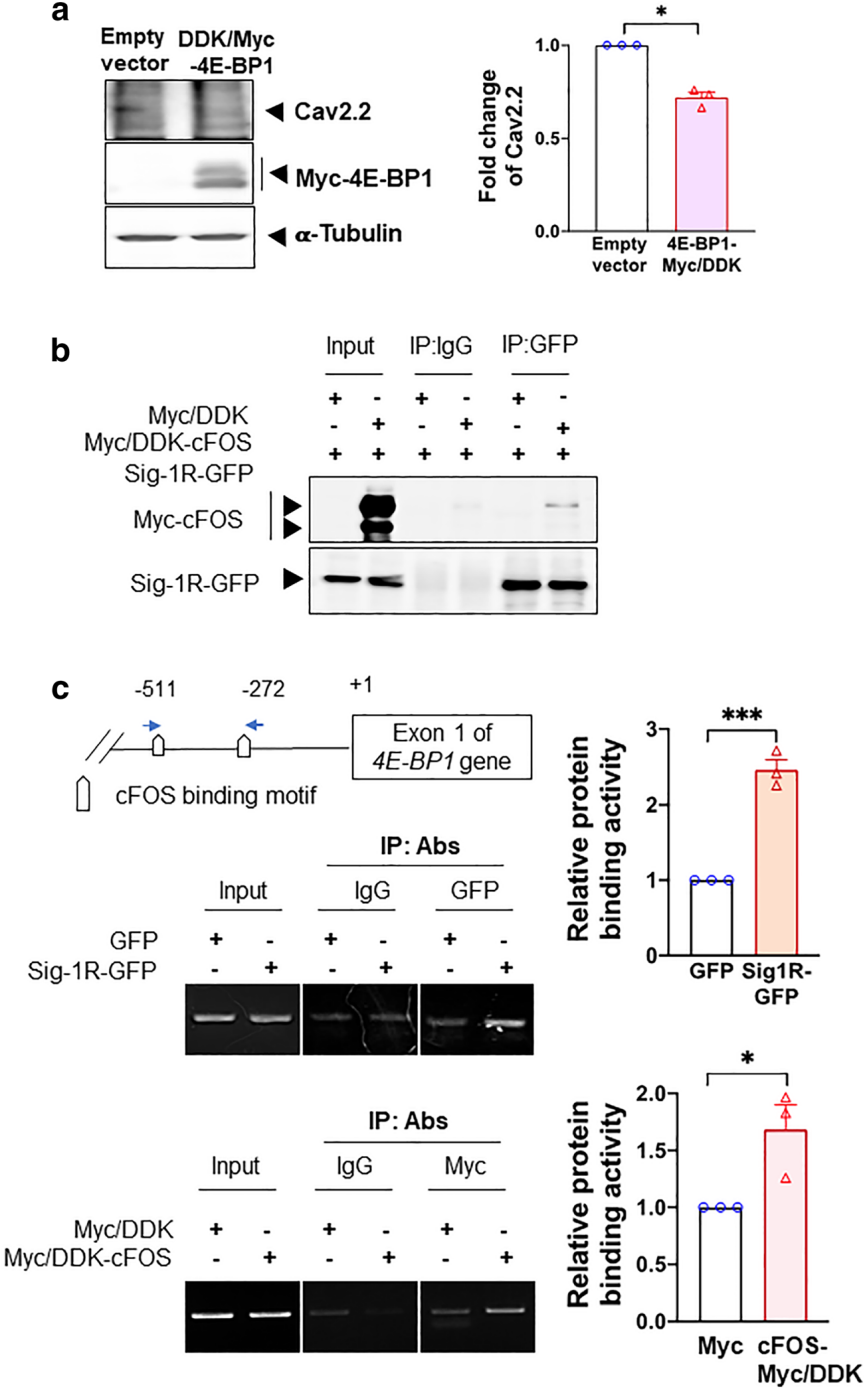
cFOS increases 4E-BP1 transcription via its interaction with Sig-1R. **a** Overexpression of 4E-BP1 decreases Cav2.2 in HEK cells. **b** Sig-1R interacts with cFOS in HEK cells. In cFOS-Myc/DDK and Sig-1R-GFP HEK co-expressing HEK cells, sample blot demonstrates the co-immunoprecipitation of those two proteins (lane 6). **c** Co-overexpressed Sig-1R and cFOS bind to the promoter of 4E-BP1. DNAs obtained from chromatin immunoprecipitation assays testing the binding of GFP-tagged Sig-1R (upper panel) or Myc-tagged cFOS (lower panel) to the 4E-BP1 promoter in HEK cells were examined. The precipitated DNA was amplified using specific primers for the 4E-BP1 promoter. Quantified results from the PCR products were normalized to input. Summary data are presented as means ± SEM; Student’s *t* test; *P < 0.05, ***P < 0.001; *N* = 3

We therefore examined if the Cav2.2 mRNA translation is controlled by the 5′cap mechanism by overexpressing 4E-BP1-Myc in HEK cells. If the Cav2.2 translation is indeed controlled by this mechanism, then an overexpression of 4E-BP1, per its binding and locking up of eIF4E, should attenuate the protein level of Cav2.2. Indeed, results show that in 4E-BP1-Myc-overexpressing HEK cells, the Cav2.2 protein level decreases (Fig. 5a).

The possibility exists then that the Sig-1R may influence the mRNA translation of Cav2.2 indirectly by affecting the initiation of the translation via the 5’cap-dependent mechanism specifically by influencing 4E-BP1 or eIF4E.

We started by examining the possibility that the Sig-1R may control 4E-BP1.

### Sig-1R Recruits cFos to the 4E-BP1 Promoter

We demonstrated previously that the Sig-1R can interact with transcription repressor [37]. Therefore, the possibility exists that the Sig-1R may regulate the transcription of 4E-BP1 by recruiting certain transcription factors to its promoter. At the transcription factor prediction website (http://alggen.lsi.upc.es/cgibin/promo_v3/promo/promoinit.cgi?dirDB=TF_8.3), a total of 6 transcription factors were identified and predicted to be at the 4E-BP1 promoter region (Supplementary Figure S6A). We overexpressed these transcription factor candidates in HEK cells and found that the 4E-BP1 mRNA level increases in cells overexpressing cFos, myeloid zinc finger 1 (MZF1), POU class 2 homeobox 1 (OCT1), and SOX2 but not CCAAT/enhancer-binding protein delta (CEBPD) and SP3 (Supplementary Figure S6B).

Among these 4 transcription factors, we decided to focus on cFos for two reasons: (1) it apparently yields slightly higher or a comparable 4E-BP1 mRNA level when compared with other factors (Supplementary Figure S6B); (2) it was reported to be upregulated after SNI [50].

We examined next if the Sig-1R may interact with cFos and, if so, may bind the promoter of 4E-BP1. We used HEK cells overexpressing Sig-1R-GFP and cFos-Myc to perform those experiments. Indeed, Sig-1R and cFos interact with each other (Fig. 5b). Further, chromatin immunoprecipitation assays show that there is an increase of binding between the 4E-BP1 promoter and Sig-1R-GFP or cFos-Myc when each protein was overexpressed (Fig. 5c). Those results suggest that the Sig-1R perhaps by working together with cFos can increase the promoter activity of 4E-BP1, thus enhancing its transcription.

We next provide evidence that the Sig-1R regulates the transcription of 4E-BP1 and the effect thereafter.

### Sig-1R Knockout Increases the Transcription of 4E-BP1: Effect on the eIF4E Binding to the Cav2.2 mRNA

**Fig. 6:**
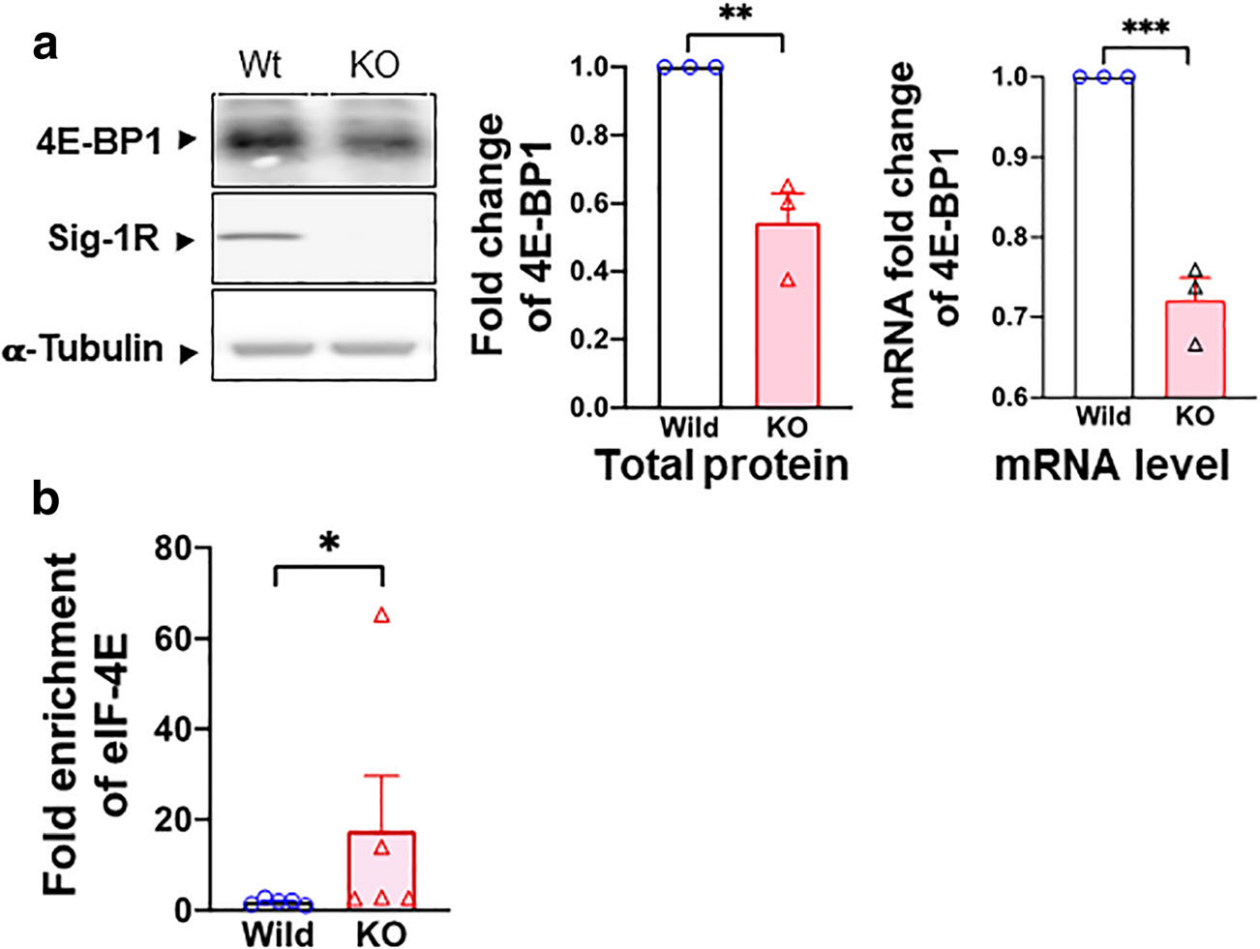
Sig-1R regulates 4E-BP1 and its effect on eIF4E. **a** Sig-1R KO decreases the transcription and translation of 4E-BP1 in HEK cells. Sample blot demonstrates a decrease of 4E-BP1 protein in Sig-1R KO HEK cells. Summary data reveal an elevated level of protein and mRNA of 4E-BP1 in Sig-1R KO HEK cells. **b** Sig-1R knockout (KO) increases eIF4E and Cav2.2 mRNA binding. In the RNA-IP test, eIF4E binding to Cav2.2 5′cap region increases in Sig-1R KO HEK cells. Data represent means ± SEM; Student’s *t* test; Mann-Whitney test for **b**; *P < 0.05, ***P < 0.001

We tested if the Sig-1R may affect the transcription of 4E-BP1 by using the Sig-1R KO HEK cells. Indeed, in Sig-1R KO HEK cells, the protein expression and mRNA of 4E-BP1 are both reduced (Fig. 6a), indicating that the Sig-1R increases the transcription of 4E-BP1.

Since 4E-BP1 is decreased in Sig-1R KO cells, 4E-BP1 should bind less of the available eIF4E as per the 5′cap-dependent translation. As a result, more eIF4E should interact with the mRNA of Cav2.2. Indeed, in Sig-1R-KO HEK cells, the binding between eIF4E and Cav2.2 mRNA increases as seen in the RNA immunoprecipitation assay (Fig. 6b).

Those results, when taken together, suggest that the Sig-1R enhances the transcription of 4E-BP1, leading to a translational inhibition of Cav2.2 via sequestration of the translation initiation factor eIF4E. We examined next the status quo of 4E-BP1and eIF4E in the rat DRG during SNI.

### cFos, 4E-BP1, and eIF4 in Spare Nerve Injury–Imposed Dorsal Root Ganglia

If physiologically relevant, what happened in cell lines as shown above should also be seen in DRGs taken from sham or SNI rats. Because of the limited quantity of samples, we focused on critical experiments to provide proofs as such.

**Fig. 7:**
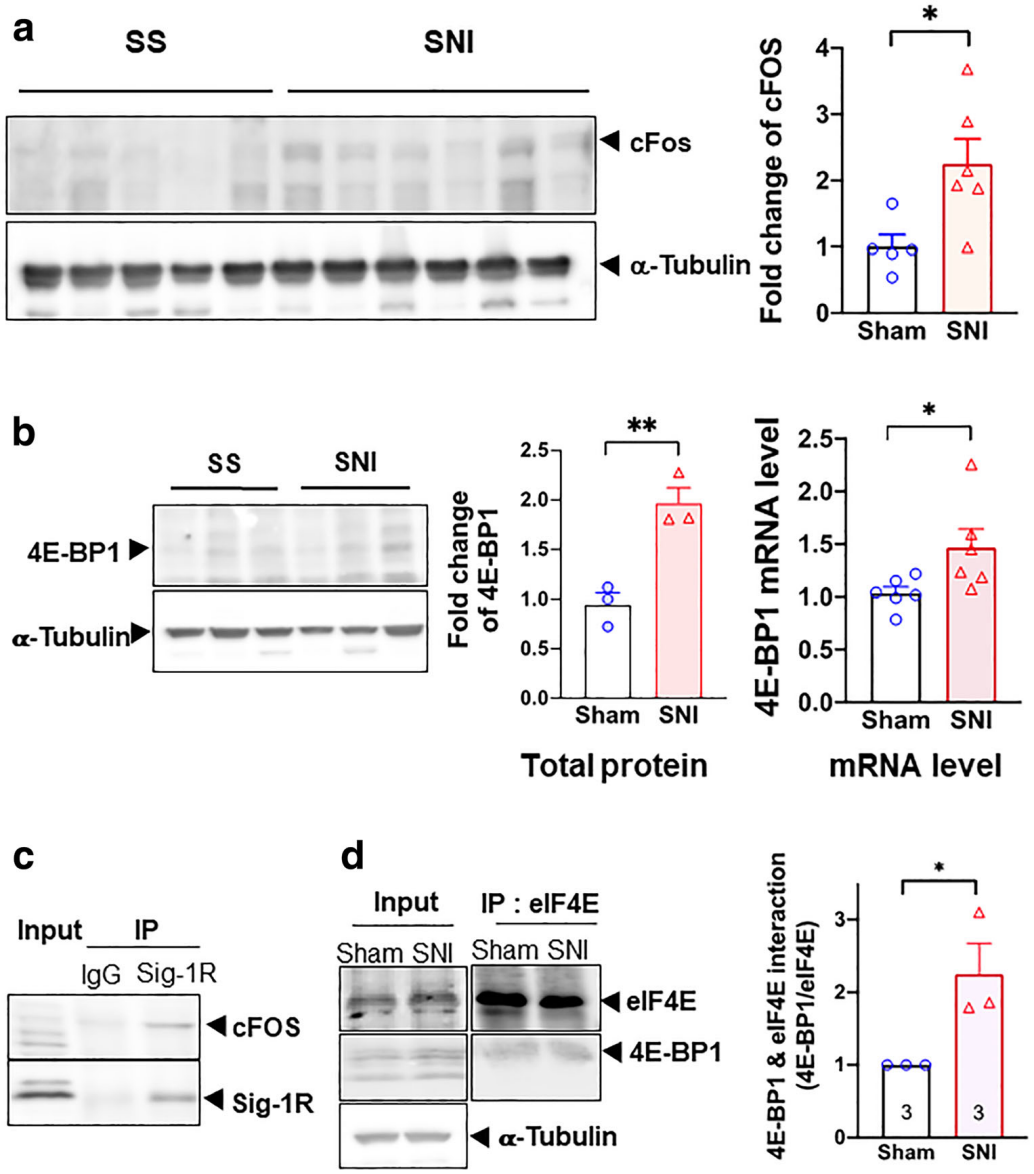
Transcriptional regulation of 4E-BP1 by Sig-1R in SNI DRG. All data here are from day 14 SNI DRGs. **a** cFOS is upregulated after SNI on day 14. All western blots are shown (left panel). Summary data with band intensity normalized to α-tubulin are on the right panel. **b** SNI increases the protein and mRNA of 4E-BP1. Western blot for protein of sham or SNI DRGs is shown on the left panel. Summary plot in the middle panel with band intensity normalized to α-tubulin. Summary plot for mRNA level from qPCR analyses is shown on the right panel. **c** Sig-1R interacts with cFOS. Blot shows immunoprecipitation (IP) with Sigma-1 receptor (Sig-1R) antibody pulling down cFOS in DRGs from SNI animals. **d** Increased interaction between 4E-BP1 and eIF4E after SNI. Representative western blot on the left panel. Summary data with band intensity normalized to eIF4E on the right panel. For western blot, each lane represents total lysate from 3 ipsilateral DRGs (right 4th to 6th lumbar DRG, L4-L6) of individual animal (sham or SNI). For IP, protein lysate was combined from 2 animals (sham or SNI) of a total of 6 ipsilateral DRGs (right L4-L6). A total of three repetitive experiments were performed for IP. Data shown are means ± SEM; *P < 0.05, **P < 0.01

Western blot signal for c-FOS protein increased after SNI in this study (Fig. 7a). This result is in agreement with previous observations [50]. We also examined the co-IP between Sig-1R and c-Fos and found that the interaction between Sig-1R and c-Fos increases by about 50% in SNI DRG when compared to sham DRG (Supplementary Figure S7).

Our hypothesis, accordingly, predicted that transcription of 4E-BP1 should increase during SNI. Indeed, 4E-BP1 mRNA and protein both increase in DRGs on day 14 after SNI (Fig. 7b), indicating an upregulation of 4E-BP1. In the co-IP assay, the Sig-1R interacts with cFos in SNI DRGs (Fig. 7c). Notably, the interaction between eIF4E and 4E-BP1 may also increase on day 14 after SNI (Fig. 7d).

Those results, when taken together, suggest that the Sig-1R at the DRG causes neuropathic pain while the inhibition of Sig-1R during SNI attenuates pain.

### DRG Excitability Increases in Rats in Response to the Sig-1R Agonist

**Fig. 8:**
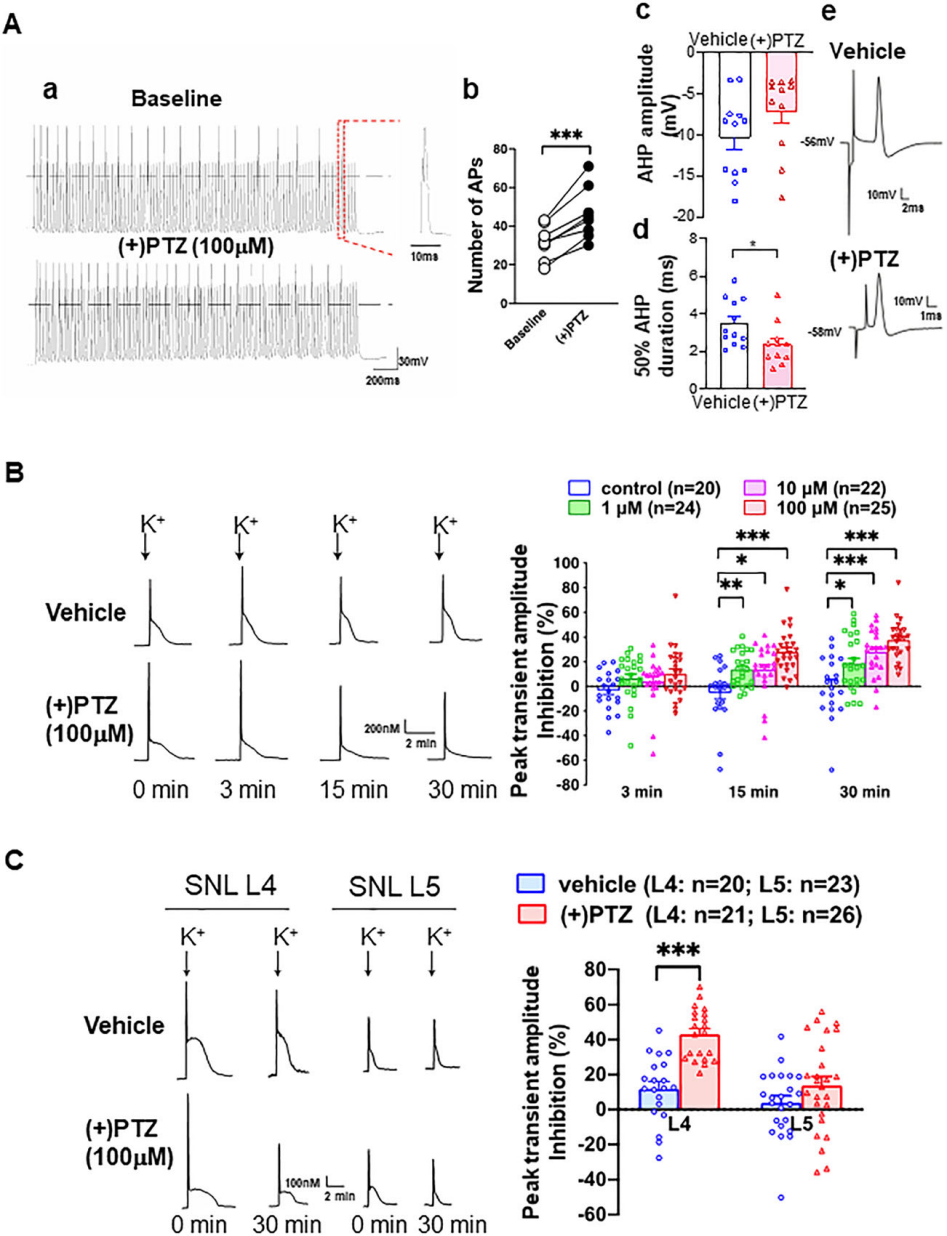
Sig-1R agonist increases neuronal excitability in sham surgery rats’ sensory neurons by inhibiting calcium influx at dorsal root ganglia. (A) (+)Pentazocine ((+)PTZ) elicits neuronal hyperexcitability in skin sham dorsal root ganglion (DRG) neurons. (Aa) (+)PTZ increases action potential (AP) generation during depolarizing stimulus trains (50 Hz for 2 s with 1 ms duration and 1.5 × threshold stimulus). Sample traces from whole-cell patch clamp show 21 APs before (+)PTZ application and 30 after. (Ab) Summary data show that (+)PTZ increases AP generation in naïve sensory neurons. (Ac) Summary data show that (+)PTZ does not affect the after hyperpolarization (AHP) amplitude. (Ad) Summary on the effect of (+)PTZ on the 50% duration of AHP. (Ae) Sample AP traces of vehicle- and (+)PTZ-treated DRG neurons were from intracellular recordings. The trace shows that (+)PTZ decreases 50% recovery of AHP duration when compared to vehicle control. AHP and duration of AHP until 50% recovery to baseline (red segment) were measured from intracellular recordings using intact DRGs and dorsal root stimulation. (B) (+)-Pentazocine inhibits K^+^-induced Ca^2+^ transient in dissociated skin-sham DRG neurons. By adding (+)PTZ (1–100 μM) to the bath solution (2 mM Ca^2+^), (+)PTZ time-dependently depresses activity-induced transient amplitude (traces). When compared with vehicle-treated cells, summary data (right panel) show that (+)PTZ dose-dependently diminishes (i.e., increased inhibition on *Y*-axis) K^+^-induced (50 mM for 3 s) Ca^2+^ transient amplitudes in skin sham DRG neurons. (C) (+)PTZ (100 μM) did not affect K^+^-induced transient Ca^2+^ amplitudes in injured neurons isolated from the spinal ligated 5th (SNL L5) DRG. In contrast, (+)PTZ administration inhibited K^+^-induced Ca^2+^ transient amplitudes in non-injured neighboring neurons from the 4th SNL L4 DRG. Note: K+-induced Ca^2+^ transient decreases in a time-dependent manner (left lower traces) in (+)PTZ-treated SNI L4 DRG neurons. Details of experimental procedures are described in the “Methods”. Data are means ± SEM; two-way ANOVA followed by Tukey’s test, Student’s *t* test or paired *t* test, *P < 0.05, **P < 0.01, ***P < 0.001

Since the excitability of sensory neurons relates to genesis of pain, the effect of the Sig-1R agonist (+)pentazocine ((+)PTZ) on the burst rate and firing of DRG was examined in dissociated DRG neurons (Fig. 8(Aa)). (+)PTZ elicits an increase in frequency of action potentials in neurons subjected to repetitive brief depolarization (Fig. 8(Ab)). Note refractory firings between action potentials in the trace. Since it is known that decreased duration of afterhyperpolarization (AHP) can increase neuronal burst rate and firing [5, 25, 51], we examined the amplitude and duration of AHP in (+)PTZ-treated DRG neurons. While (+)PTZ-treated neurons do not exhibit differences in the AHP amplitude (Fig. 8(Ac)), (+)PTZ-treated neurons show a significant decrease in the duration of AHP (Fig. 8(Ad)). The AHP duration is defined as 50% of time required to return from the AHP maximum to the resting membrane potential. Sample trace from vehicle- or (+)PTZ-treated neuron is shown in Fig. 8(Ae). When taken together, those results suggest that the activation of Sig-1R causes hyperexcitability of DRG neurons.

The duration of AHP is regulated by the activity of calcium-activated potassium channels (K_Ca_) [52, 53], which in turn depend on the inward calcium current (*I*_Ca_). Indeed, (+)PTZ has been reported to cause a decrease of *I*_Ca_ [28]. The decrease of *I*_Ca_ in DRG neurons after peripheral injury has been shown to be due to the decrease of *I*_Ca_ through VGCCs at the DRGs [25, 54]. We speculated therefore that the increase of neuronal excitability caused by the activation of Sig-1R is mediated by the inhibition of VGCCs.

We therefore examined the depolarization-induced transient elevation of [Ca^2+^]_c_ to determine if Sig-1R activation is accompanied by a reduced [Ca^2+^]_c_ transient. (+)PTZ indeed depresses the depolarization-induced transient amplitude in a concentration and time-dependent fashion (Fig. 8(B)). To selectively examine the effect of axonal injury, we used the 5th lumbar (L5) spinal nerve ligation (SNL) neuropathic pain model, which showed that axotomy (L5 neurons) reduces transient amplitude of the dissociated somata compared to those of the intact adjacent L4 neurons (left panel, Fig. 8(C)). Interestingly, (+)PTZ, which strongly inhibits the peak [Ca^2+^]_c_ in intact L4 neurons, does not further decrease peak [Ca^2+^]_c_ in the L5 neurons (shown as % peak inhibition, right panel, Fig. 8(C)), suggesting that axotomy has altered the VGCCs. The near elimination of transients in Ca^2+^-free bath solution in both vehicle-treated neurons (89.9 ± 3.0% reduction, *n* = 5) and neurons treated with (+)PTZ (100 μM, 95.0 ± 0.9%, *n* = 8, Figure S3A) confirms the dependence on Ca^2+^ entry through VGCCs.

It has been shown that the calcium-induced calcium release (CICR) from ER stores can amplify depolarization-induced transients in sensory neurons [55]. Accordingly, we tested the action of Sig-1R on CICR by using the ryanodine receptor blocker dantrolene (10 μM). Dantrolene reduces the depolarization-induced [Ca^2+^]_c_ by 79.0 ± 1.6% in vehicle-treated neurons, indicating a substantial contribution of CICR to the Ca^2+^ transient (Figure S8B; left panel). This dantrolene effect is not altered by co-administration of Sig-1R agonists ((+)PTZ, 75.7 ± 1.7% reduction; Figure S8B; right panel). Those results suggest that the predominant pathway of the Sig-1R regulation on activity-related changes in [Ca^2+^]_c_ in uninjured sensory neurons is through the modulation of VGCCs rather than CICR.

Taken together, those data above suggest that the Sig-1R-induced sensory hypersensitivity in naïve animals is mediated by the inhibition of calcium influx which in turn leads to the neuronal hyperexcitability of the sensory neurons. The first portion of the results (Fig. 1–Fig. 7) supports this suggestion.

### Intra-DRG Injection of Sig-1R Agonist in Naïve Rat Causes Pain While the Antagonist Attenuates SNI-Induced Pain

**Fig. 9:**
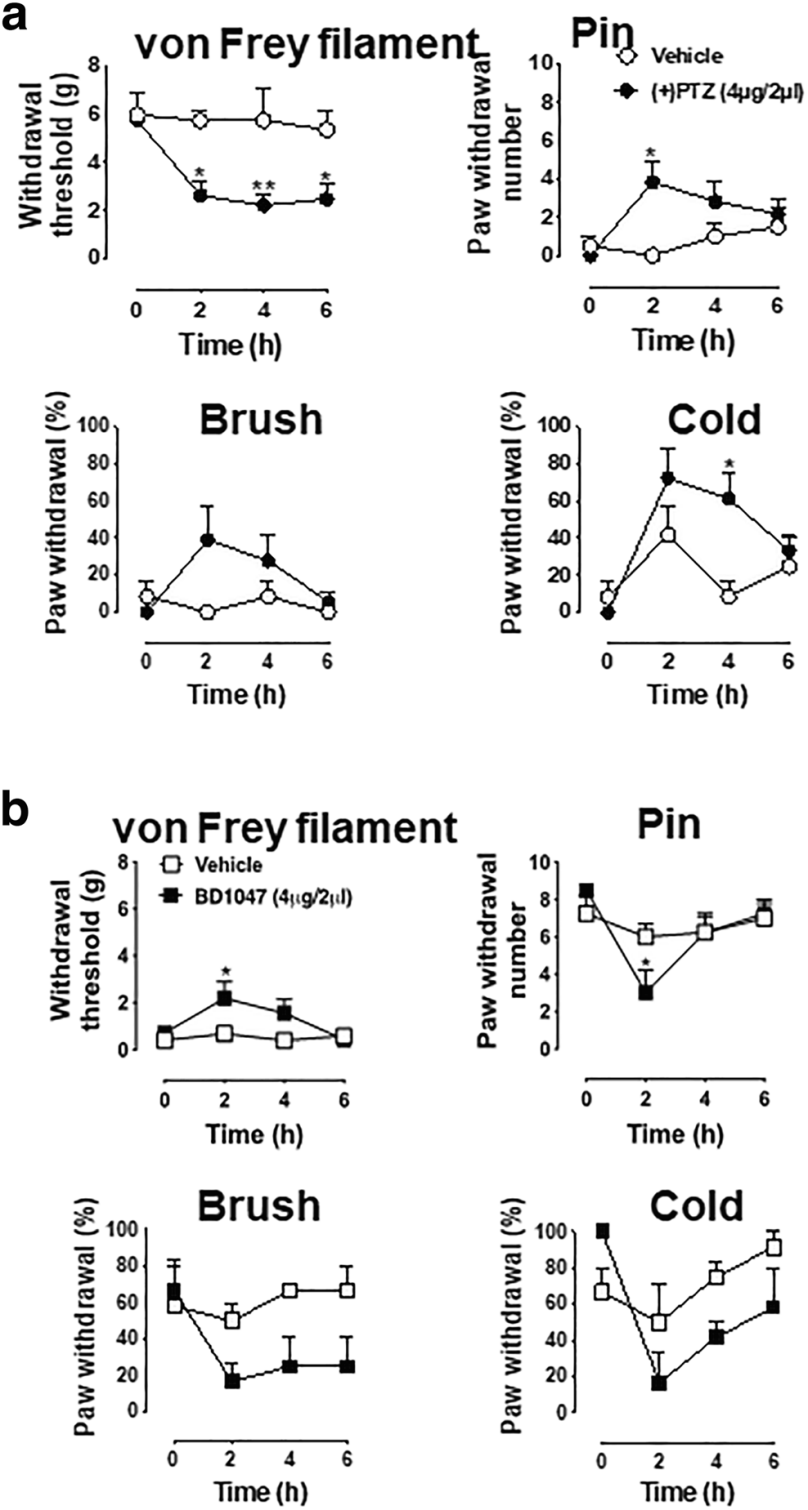
Blocakade of Sig-1R attenuates SNI-induced pain responses whereas Sig-1R agonist elicits hyperalgesic responses in naive animals. **a** (+)-Pentazocine produces hypersensitivity in naïve rats. Sig-1R agonist (+)-pentazocine ((+)PTZ)) decreases mechanical withdrawal threshold and increases response rates to mechanical and cold stimuli in naive rats. Detailed information of animal, surgery procedure, and sensory tests are described in the Methods. **b** BD1047 attenuates hyperalgesic responses in SNI rats. The sensory tests of threshold mechanical stimulation (von Frey filaments), noxious stimulation (pin), soft touch (brush), and cold chemical stimulation (acetone drop) following intraganglionic injection of Sig-1R antagonist (BD1047, 4 μg/2 μl) in SNI rats were evaluated in the plantar skin of the paw ipsilateral with the injected DRG. BD-1047 reduces mechanical and thermal hypersensitivity. Data are means ± SEM; *n* = 4 in each group except (+)PTZ-injected naïve rat group (*n* = 6); two-way ANOVA followed by Tukey’s test, *P < 0.05, **P < 0.01

Of course, we cannot examine the effect of the Sig-1R agonist in SNI rats because they are in pain already. We examined therefore if there is constitutive regulation of sensory neuron function [30] by the Sig-1R by injecting the Sig-1R agonist into the DRG in sham-operated rats. (+)PTZ produces a decrease of withdrawal threshold from threshold mechanical stimuli during von Frey testing in naïve rats (Fig. 9a), and increases the frequency of response to cold stimulation by acetone, and hyperalgesia responses to pin (Fig. 9a).

The Sig-1R antagonist BD1047 decreases heightened tactile sensitivity (allodynia) significantly in von Frey filament threshold testing and increases the frequency of hyperalgesia-type responses to noxious mechanical stimulation by pain (Fig. 9b). Though statistically nonsignificant, a trend of decline of responses to brush and cold stimuli are seen in BD1047-treated SNI animals when compared to controls (Fig. 9b). These findings are consistent with the view that knockout or systemic blockade of Sig-1R diminishes the peripheral nerve injury–induced hypersensitivity [20, 21].

## Discussion

Hyperexcitability of injured DRG neurons is attributable at least in part to reduced calcium influx and the associated decrease of intracellular Ca^2+^ transient [25, 56] that in turn fails to activate Ca^2+^-activated potassium channels [51]. We show here that the Sig-1R, a dynamic ER molecular chaperone, can regulate the DRG excitability and confer pain by regulating VGCCs at the genomic and cellular biology levels and demonstrate for the first time that the direct injection of Sig-1R antagonist into DRG is a clinically feasible route for the treatment of neuropathic pain.

Our results showing that SNI causes a reduction of Cav2.2 but not Cav1.2 is at variance with some previous reports. For example, although in this study we did not observe a significant change of Cav1.2 protein or mRNA after SNI, the Cav1.2 protein expression and mRNA level were reported to decrease after sciatic nerve ligation [57]. A decrease of Cav1.2 mRNA level was found after 7 days in a chronic constriction injury model [58]. Also, an increase of Cav2.2 at DRGs after the tibial injury was reported [59]. We do not know at present whether the difference reflects different types of surgical injury employed or due to other unknown factors. However, our results are consistent with other reports showing that the Cav2.2 mRNA level was not changed after nerve injury [60, 61] and Cav2.2 is downregulated in the injured 5th lumbar (L5) ganglion [62].

Our finding here that the Sig-1R may translocate from ER to the nuclear envelope, assisted by the protein transport protein Sec61β may explain how the Sig-1R moves from ER to the nuclear envelope in our previous report [37]. It is interesting to note that the Sig-1R at the nuclear envelope can associate with nuclear inner membrane protein emerin to recruit chromatin remodeling factors resulting in Sp3 binding to the MAOB promoter [37]. However, in the present study, we found that the Sig-1R can recruit yet another transcription factor cFOS (Fig. 5), resulting in the gene activation of 4E-BP1. Thus, it appears that the Sig-1R may recruit different transcription factors to regulate the transcription of different genes. How these two ways operate apparently opposite signaling pathways is unknown. We notice however that the repressive transcription of MAOB gene is initiated by the activation of Sig-1R by cocaine [37], whereas the enhanced transcription of 4E-BP1 in the present report is initiated by the SNI. One can only speculate that cocaine and SNI cause Sig-1Rs to interact with different nuclear envelope partners with different transcriptional output. The speculation remains to be clarified in the future.

The proposed mechanism in this study whereby the Sig-1R attenuates the Cav2.2 mRNA translation by increasing 4E-BP1 to sequester and prevent eIF4E from binding to the mRNA poses two important points: (1) Since Cav1.2 mRNA and protein levels are not affected by SNI, as shown in this study, is Cav1.2 translation not 5’cap-dependent? (2) The peripheral nerve injury procedure utilized in this study must reduce the translation of many proteins since the 5′cap-dependent mechanism controls translation in general or at least in part. The answer to the first question is: yes. The Cav1.2 translation is eIF2α-dependent and may not be 5′cap-dependent [63]. The answer to the second question is “yes” as well. Employing a ribosomal profiling technique, Uttam and his colleagues [64] identify 31 genes whose proteins are downregulated after SNI in mouse DRGs. Among those 31 genes, 29 genes such as TSK3 [65] show no change of their mRNA level suggesting an altered translation.

The apparent decrease of Cav2.2 at the plasma membrane (Fig. 2b) of SNI DRGs would reduce the influx of calcium thus leading to neuronal hyperexcitability. Thus, reduced translation of Cav2.2 combined with its apparent decrease at the plasma membrane may play a role in the genesis of DRG neuronal hyperexcitability. However, since the Sig-1R is a pluripotent modulator [66], its effect on hyperexcitability or neuropathic pain may relate to its action on other molecular targets as well. For example, potassium channels and their epigenetic modifications at the DRG have been shown to relate to neuropathic pain [67]. Whether the Sig-1R affects SNI-induced pain via potassium channels certainly warrants investigation in the future. The same applies to the potential action of Sig-1R on NMDA receptors [20]. It has to be mentioned here that we are not claiming the Cav2.2 is the only molecule responsible for SNI neuropathic pain. Nor do we claim here that the translational regulation of Cav2.2 is the sole mechanism regulating Cav2.2 as it relates to SNI neuropathic pain. Since Cav2.2 protein levels can be regulated via endocytosis, turnover, or extra-synaptic re-localization, its dynamic regulation is not solely restricted to the translational regulation.

The Sig-1R apparently acts directly or indirectly as a transcription factor in partnership with cFos in this study. However, whether the Sig-1R per se acts as a transcription factor by itself is unclear at present. More studies are needed in the future.

The direct injection of Sig-1R antagonist into DRGs, as shown here, represents a possible clinical approach for treating neuropathic pain [7]. Targeting Sig-1Rs to reduce pain would provide a non-opioid alternative to pain treatment treatment.

We suggest that the direct intra-DRG application of Sig-1R antagonist(s) is a very tangible way to attenuate neuropathic pain. Not only is the procedure a clinical norm but also several of the Sig-1R antagonists are clinically used “old” drugs waiting to be repurposed for neuropathic pain. Relevant Sig-1R antagonists include haloperidol and progesterone [9, 68, 69]. It has to be mentioned that within the context of this study, it would be desirable to demonstrate that the Sig-1R antagonists should attenuate the DRG neuronal excitability by attenuating Ca^2+^ influx in SNI rats. Indeed, knocking down Sig-1Rs in SNI DRG neurons causes a reduction of action potential frequency [70]. This result indirectly suggests that a Sig-1R antagonist would reduce Ca^2+^ influx, thus attenuating neuronal excitability. Of course, experiments are needed to test this speculation.

## Supplementary Information

ESM 1 (DOCX 16.5 mb).

## Data Availability

All raw data and materials are available upon reasonable request to Tsung-Ping Su.

## Abbreviations

Sig-1R: Sigma-1 receptor
DRG: dorsal root ganglion
PTZ: (+)pentazocine
MAM: mitochondria-associated endoplasmic reticulum membrane
VGCC: voltage-gated calcium channel
SNI: spared-nerve injury
CICR: calcium-induced calcium release
KO: knockout
AHP: afterhyperpolarization
HEK cell: human embryonic kidney cell
N2a: mouse neuroblastoma Neuro-2a cell
IP: immunoprecipitation
