# Correction to: Genomic Action of Sigma-1 Receptor Chaperone Relates to Neuropathic Pain

**DOI:** 10.1007/s12035-021-02310-3

**Published:** 2021-02-01

**Authors:** Shao-Ming Wang, Nino Goguadze, Yuriko Kimura, Yuko Yasui, Bin Pan, Tzu-Yun Wang, Yoki Nakamura, Yu-Ting Lin, Quinn H. Hogan, Katherine L. Wilson, Tsung-Ping Su, Hsiang-en Wu

**Affiliations:** 1 Cellular Pathobiology Section, Integrative Neuroscience Research Branch, Intramural Research Program, National Institute on Drug Abuse, NIH/DHHS, Suite 3512, 333 Cassell Drive, Baltimore, MD 21224 USA; 2 Department of Anesthesiology, Medical College of Wisconsin, Milwaukee, WI 53226 USA; 3 Department of Psychiatry, College of Medicine, National Cheng Kung University, Tainan City, 70101 Taiwan; 4 Department of Pharmacology, Graduate School of Biomedical & Health Science, Hiroshima University, Hiroshima, 734-8553 Japan; 5 Department of Cell Biology, Johns Hopkins University School of Medicine, Baltimore, MD 21205 USA

**Correction to: Mol Neurobiol**

10.1007/s12035-020-02276-8

The original version of this article unfortunately contained some mistakes. The current Electronic Supplementary file is not correct and should be updated.

The original paper has been corrected.

