# Supplementary material for: Genomic Action of Sigma-1 Receptor Chaperone Relates to Neuropathic Pain
Source: Mol Neurobiol. 2021 Jan 18;58(6):2523–41. doi: 10.1007/s12035-020-02276-8 (PMC8128747; doi:10.1007/s12035-020-02276-8)
Supplement: Supplementary file 1 — (DOCX 16.5 mb). [file 12035_2020_2276_MOESM1_ESM.docx]

Fig. S1


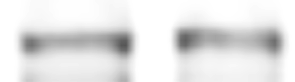

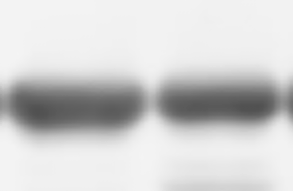

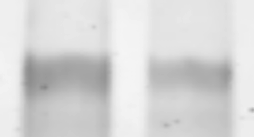

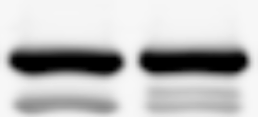

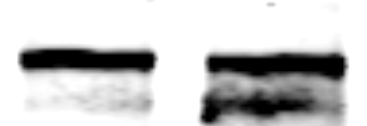

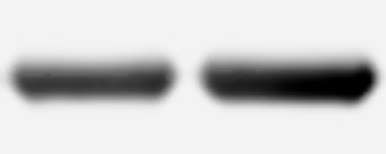


**(A)**

**3 days**

**14 days**

Sham SNI

Sham SNI

Sham SNI

**28 days**

**total protein**

**mRNA level**

**(B)**

Cav1.2

α-tubulin

**Figure S1. SNI does not change the Cav1.2 transcription or translation.** (**A**) Representative western blots of Cav1.2 protein expression at different time points after SNI in rat DRGs. α-Tubulin served as internal control. (**B**) Summary data show no alteration on the level of Cav1.2 protein or its mRNA after SNI. Data are means ± SEM; Two-way ANOVA.

**Sig-1R-GFP**


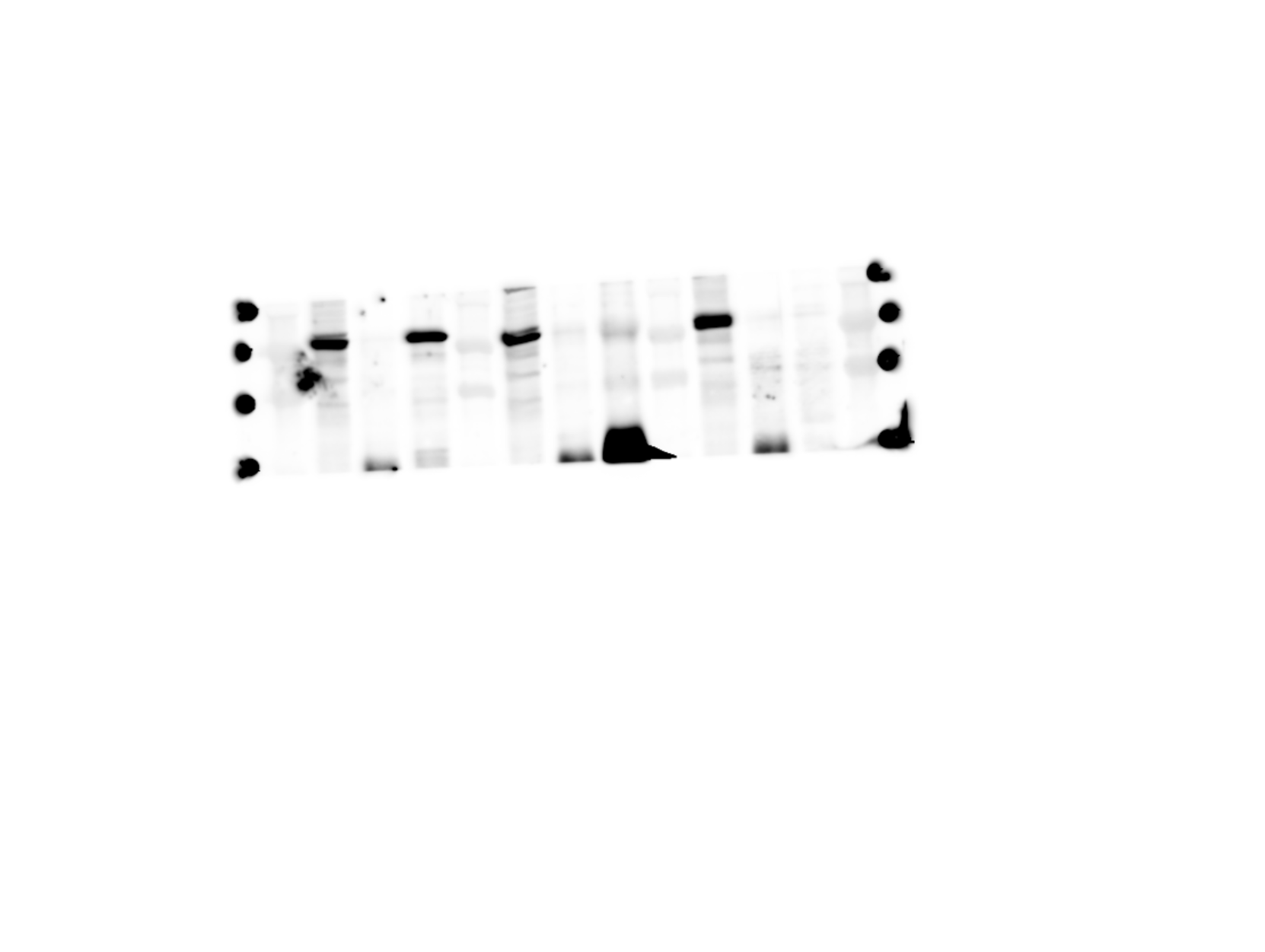


**Input**

**IgG**

**GFP**

**IP**

**Input**

**IgG**

**Sec61𝛃**

**IP**

**Sig-1R-GFP**

**Sec61𝛃**


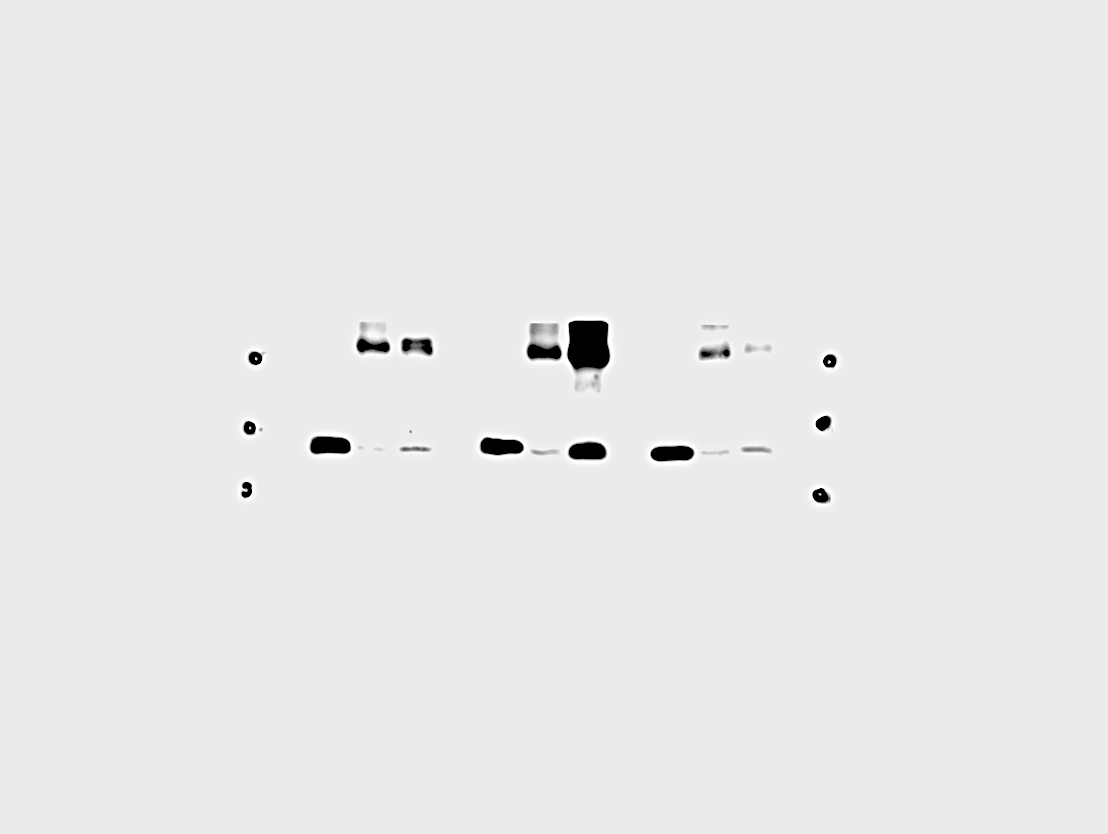

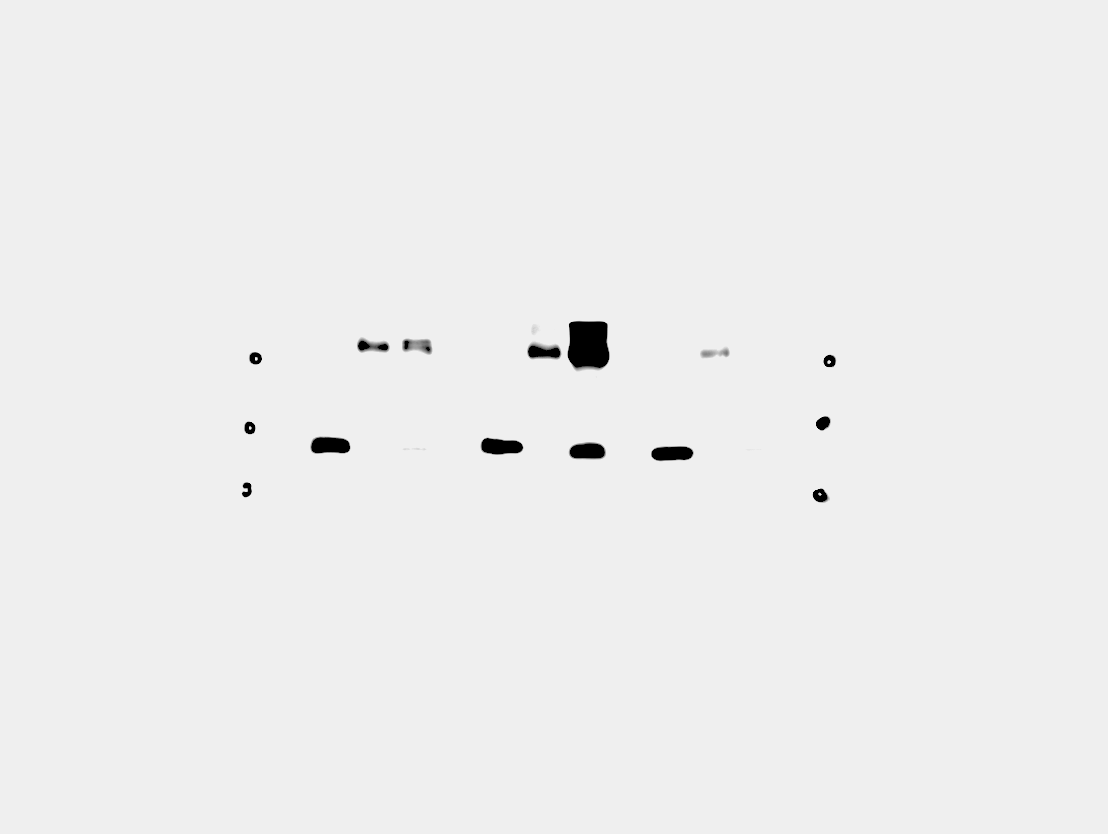

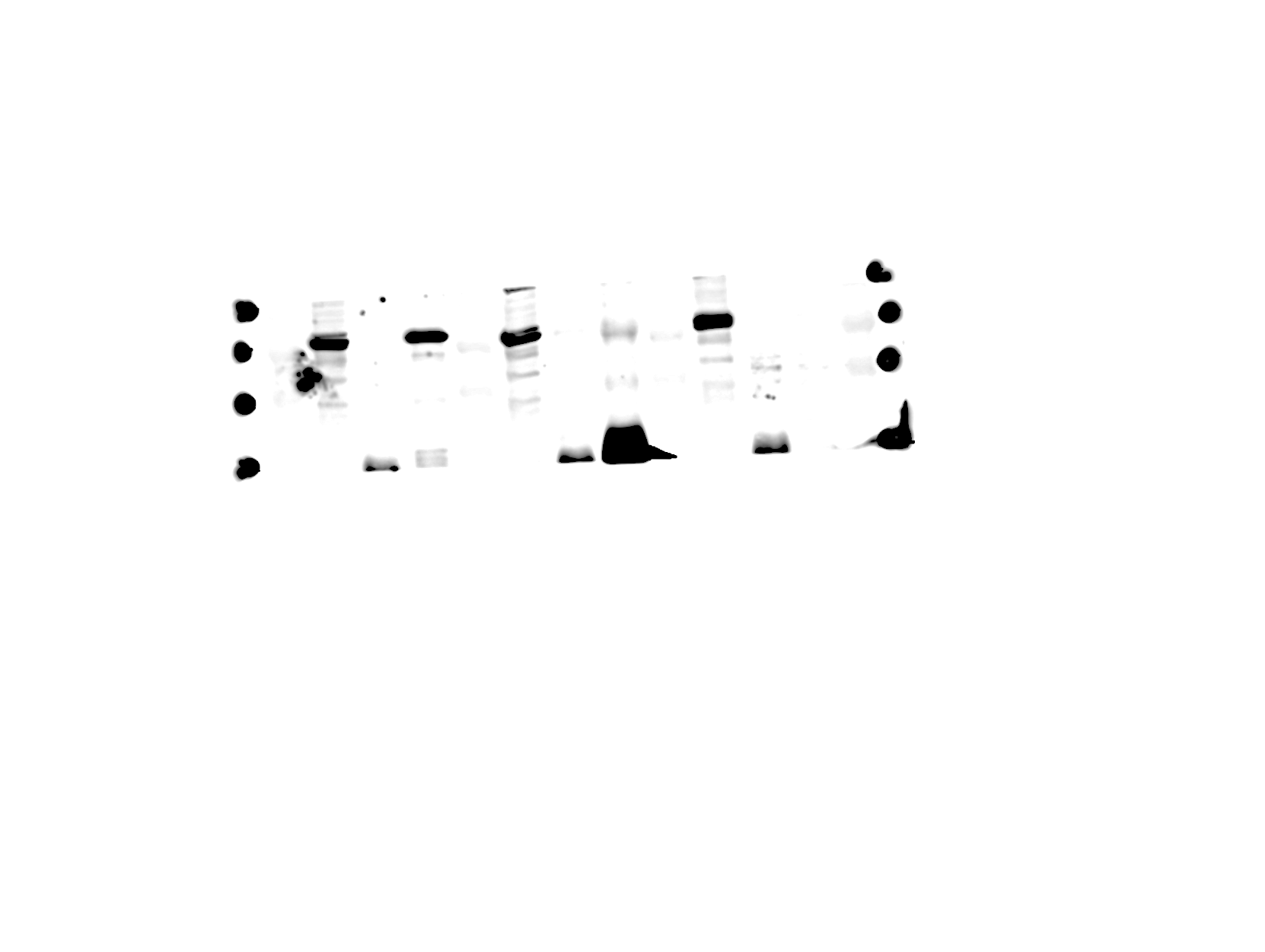


Fig. S2

**Figure S2. Sigma-1R interacts with endogenous Sec61β in N2a cells.** In N2a cells, Sig-1R-EYFP was overexpressed. Immunoprecipitation with GFP or Sec61β antibodies show an interaction between Sigma-1R (Sig-1R) and Sec61β.

**HA-Sec61𝛃**

**Sig-1R**


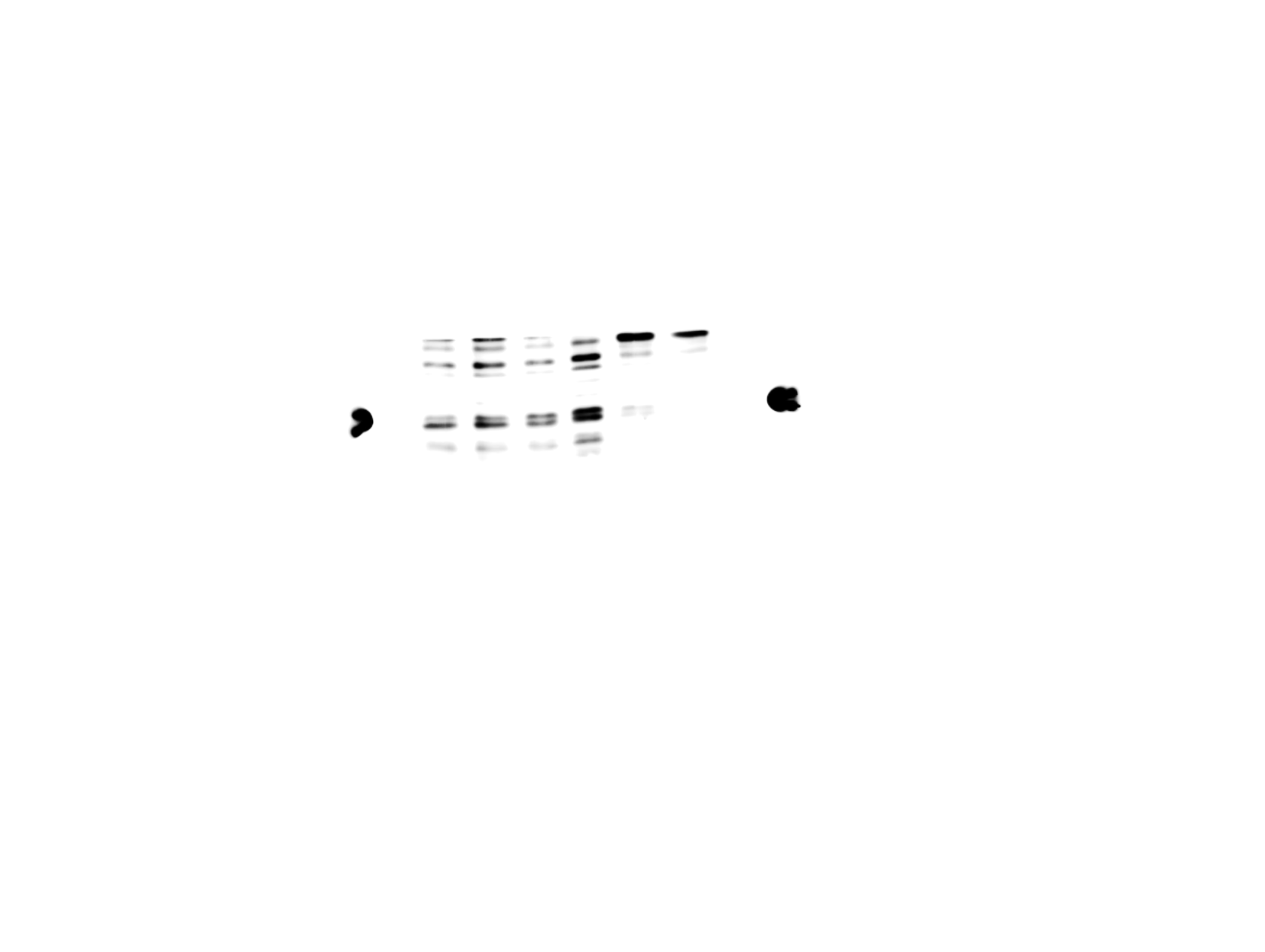

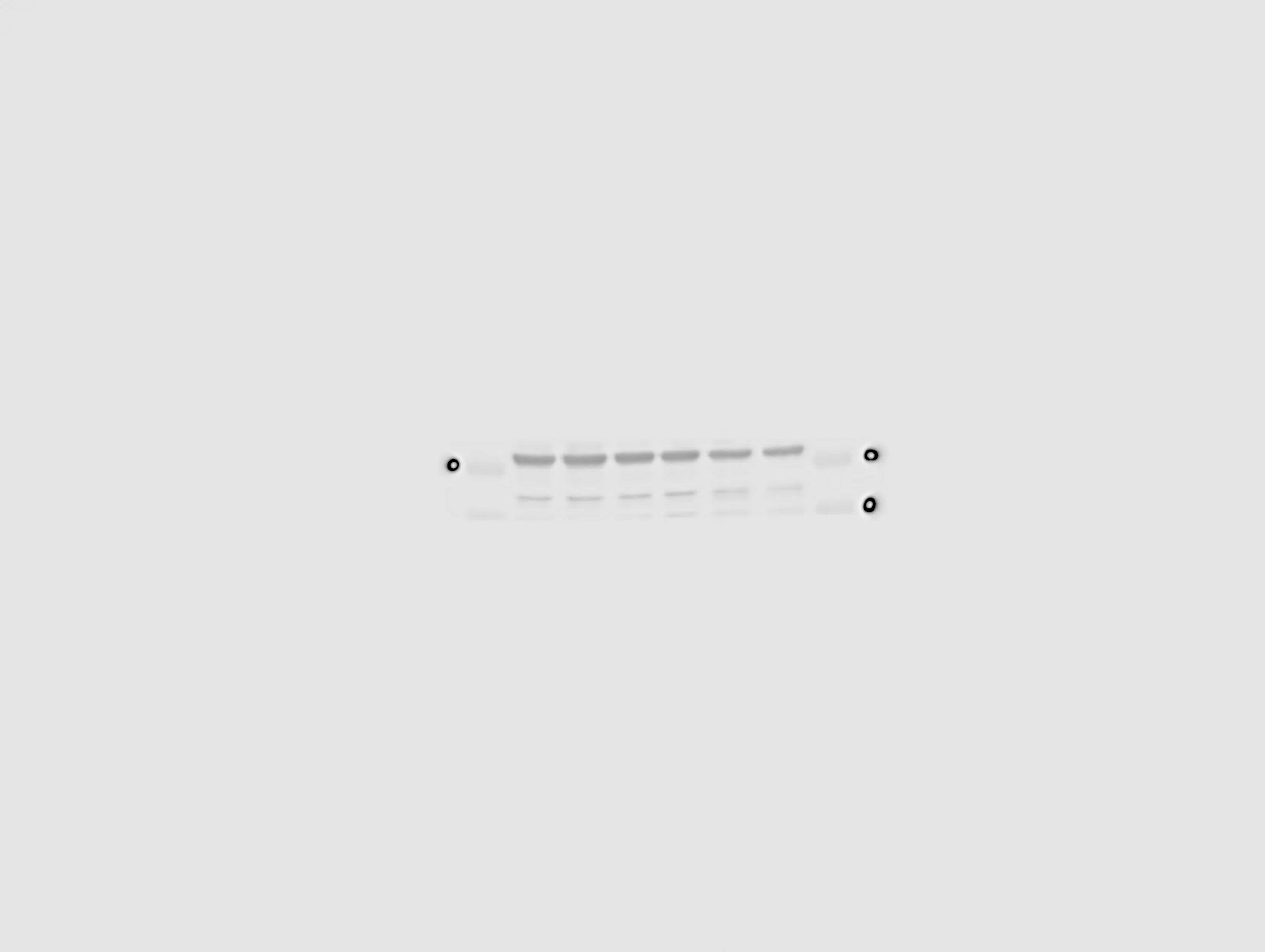


**HA-Sec61 𝛃**

**Total lysate**

**HA**

**+**

**-**

**-**

**+**

**𝛂-Tubulin**

**Sig-1R/𝛂-Tubulin ratio**

**1.00**

**1.25**


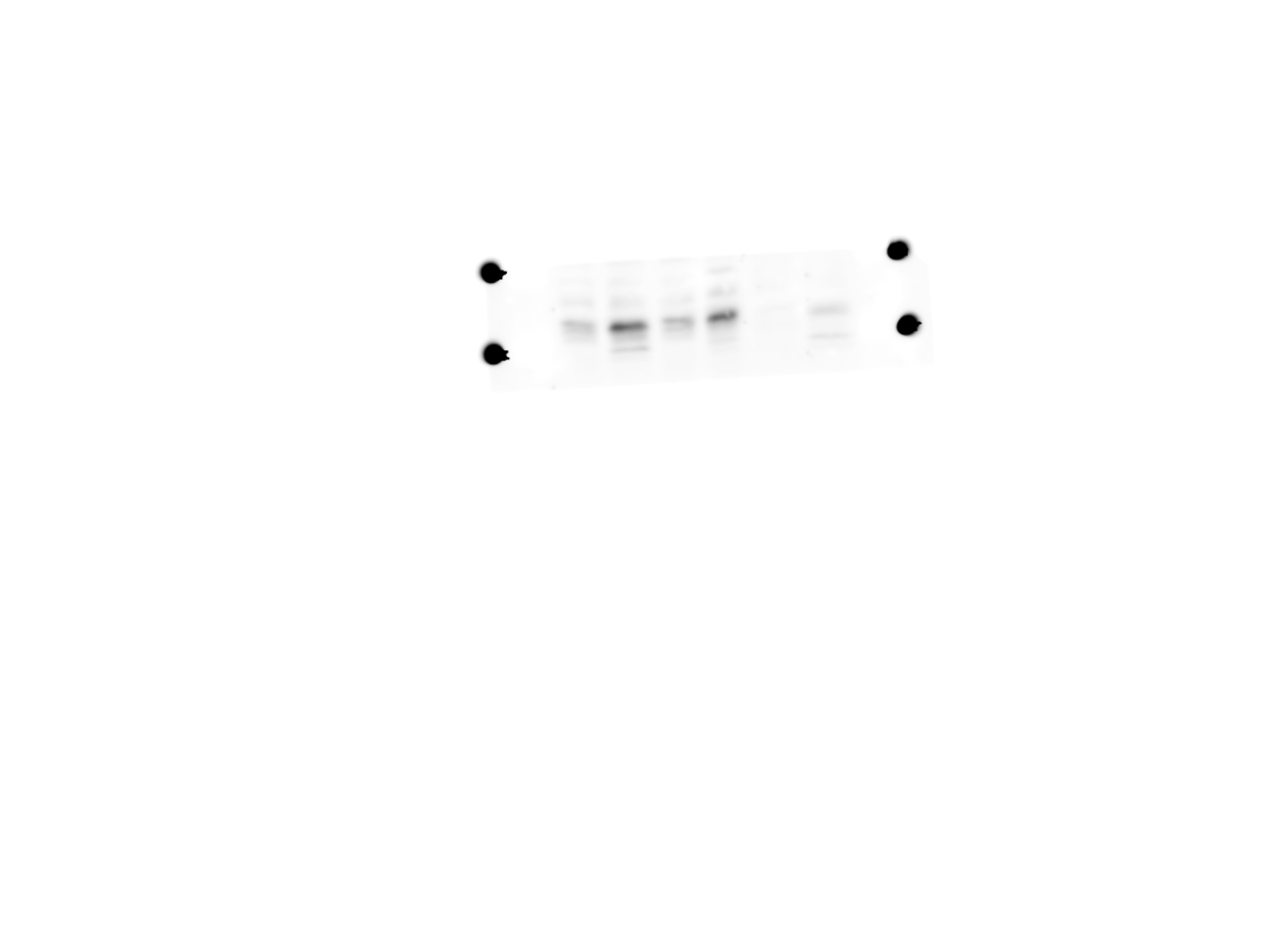


Fig. S3

**Figure S3. HA-Sec61β overexpression upregulates sigma-1R proteins in N2a cells.** In N2a cells, HA-Sec61β was overexpressed. Western blotting shows an increase of Sigma-1R (Sig-1R) protein level in total cellular lysates of HA-Sec61β-expressing N2a cells.


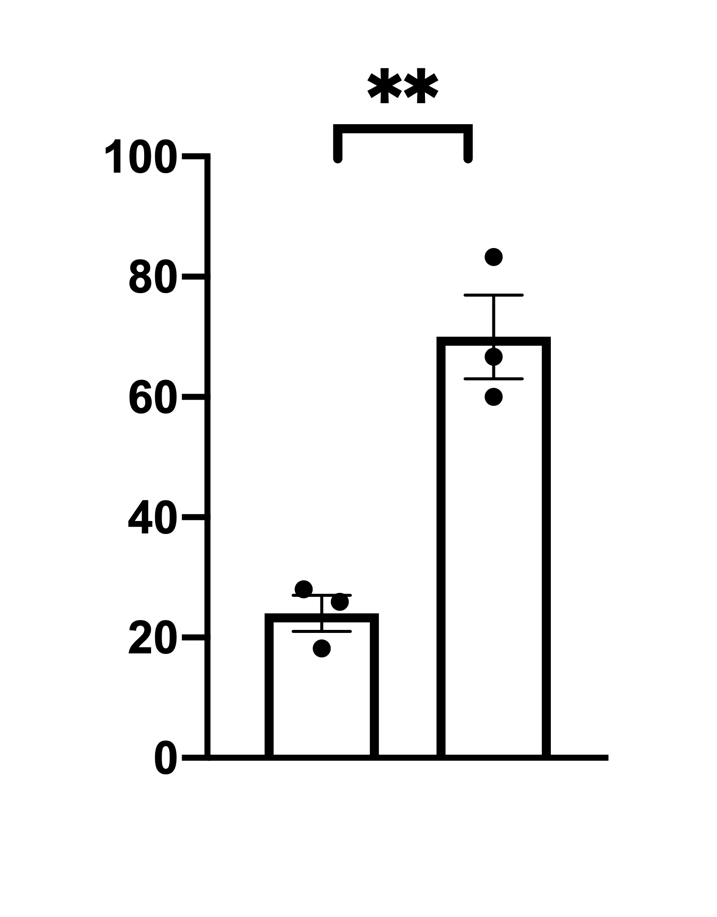


**HA**

**HA-Sec61β**

**% of Sig-1R-GFP colocalization**

**with Emerin**

Fig. S4

**Figure S4. Quantification of the Sig1R-GFP colocalization with Emerin in HA-Sec61β-overexpressing N2a cells.** Summary data on N2a cells transfected with HA or HA-Sec61β and Sig-1R-GFP. Sixty-three cells in three cover slips containing HA-transfected cells were examined for positive co-localization. Percentage of positive cells in each cover slip was calculated. Likewise, percent positive cells in each of the three cover slips containing HA-Sec61β-transfected cells (total of 54 cells) were calculated. Data are means ± SEM; unpaired Student t-test; ***p* < 0.01.


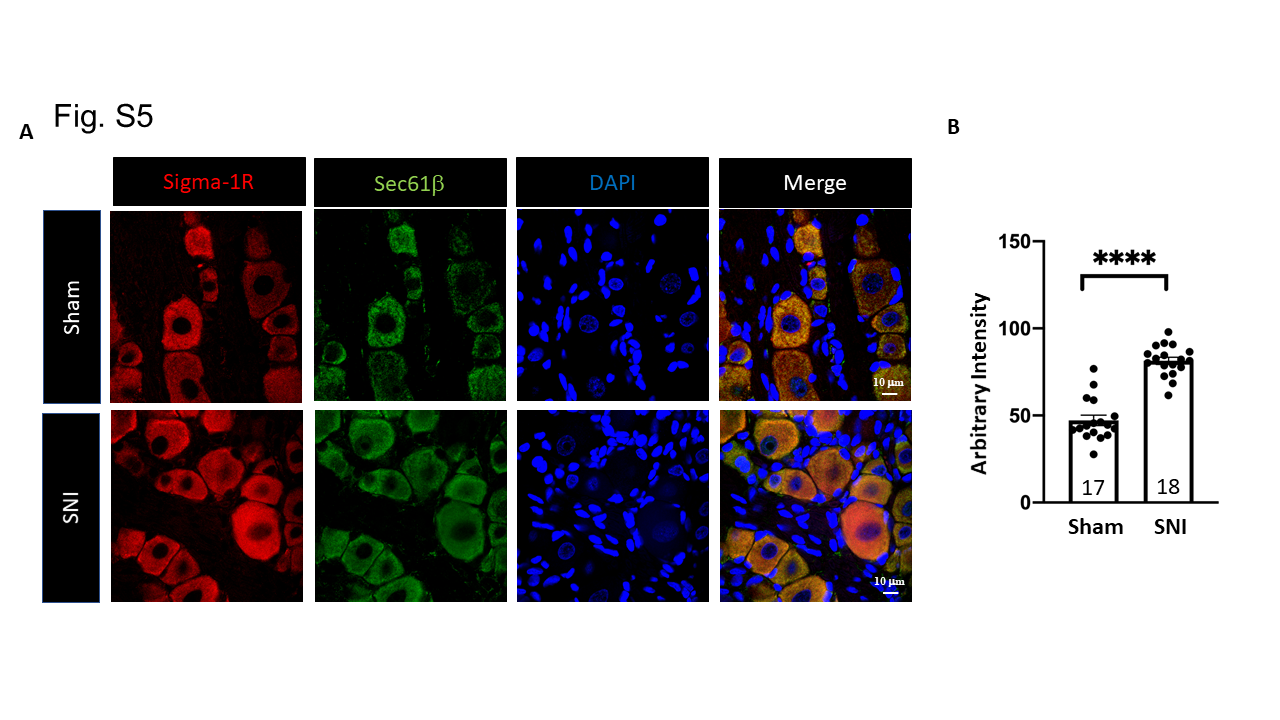


**Figure S5.** **Increased colocalization between endogenous** **Sig-1R and endogenous Sec61β after SNI in DRG neurons**. (A) Confocal images show clear localization of Sig-1R (red), Sec61β (green) and nuclear maker 4 ́-6-diamidino-2-phenylindole (DAPI; blue) in respective places in the Sham or SNI rat DRGs. (B) Summary data on neurons which demonstrate colocalized intensity of Sig-1R and Sec61β in skin sham or SNI DRG. The semi-quantification of the colocalization of Sig-1R and Sec61β were performed by using NIH Image J. (version 1.51b) which generates artificial intensity unit that was used in the Y-axis of the plot. The whole area of a cell, except the nucleus, was estimated for the co-localization intensity since Sig-1R and Sec61β are present in most part of a cell. Note: 17 neurons from 3 sham animals were examined; 18 neurons from 3 SNI animals were examined. Data are means ± SEM; Student t-test; *****p*<0.0001.

**SOX2**

**SP3**

**MZF1**

**OCT1**

**CEBPD**

**cFOS**

***4E-BP1***

+1

-1000

**B**

**A**

Fig. S6

**Figure S6. Predicted transcription factors at the 4E-BP1 promotor and verification of their activity in HEK cells. (A)** Diagram illustrates the 4E-BP1 promoter region and its predicted transcription factor binding motif. **(B)** Verification of promoter activation. Overexpression of cFOS, MZF1, OCT1, or SOX2 increases 4E-BP1 transcription in HEK cells. GAPDH served as internal control. Data are means ± SEM; Student *t*-test; **P* < 0.05, ****P* < 0.001.


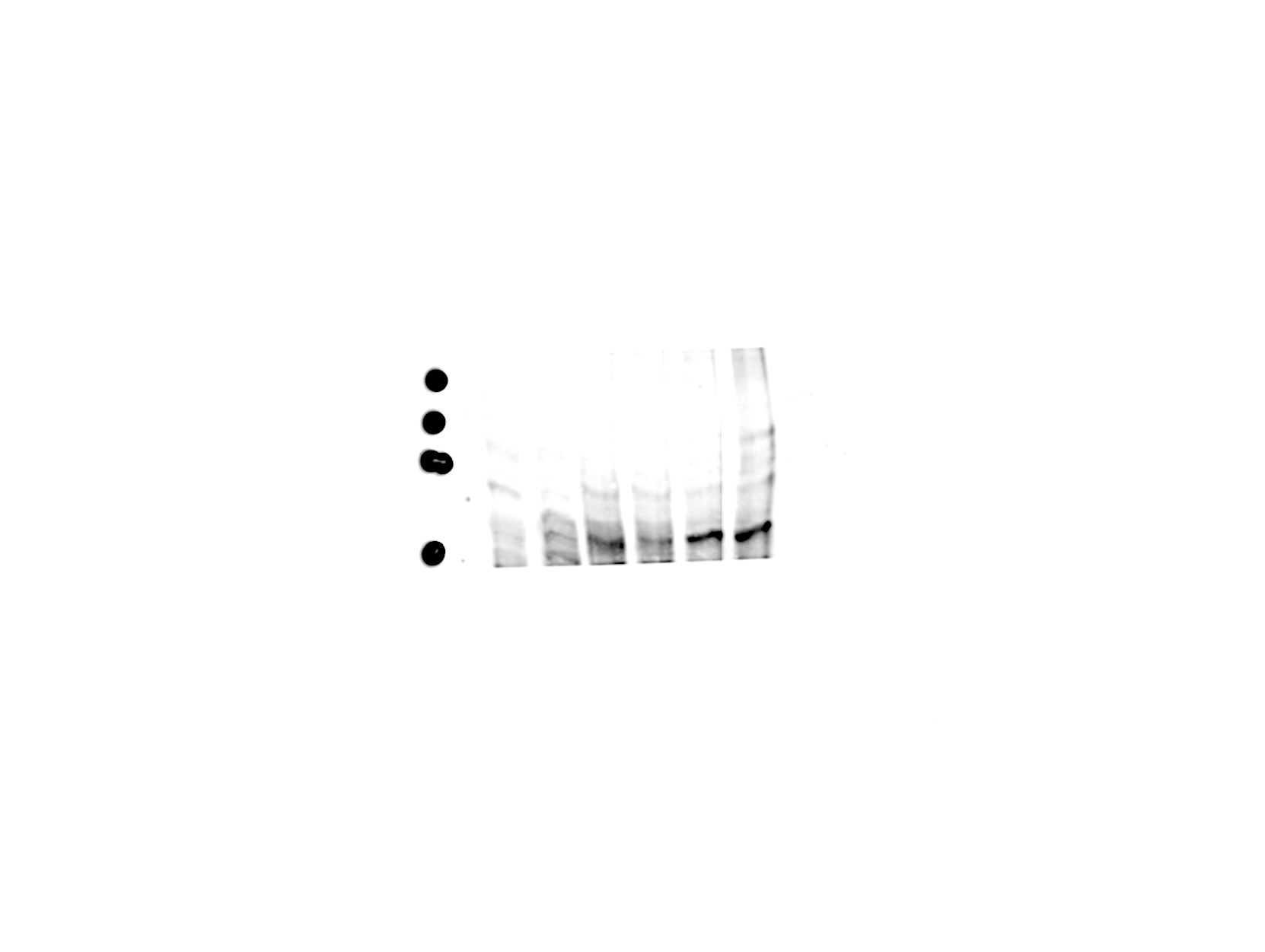

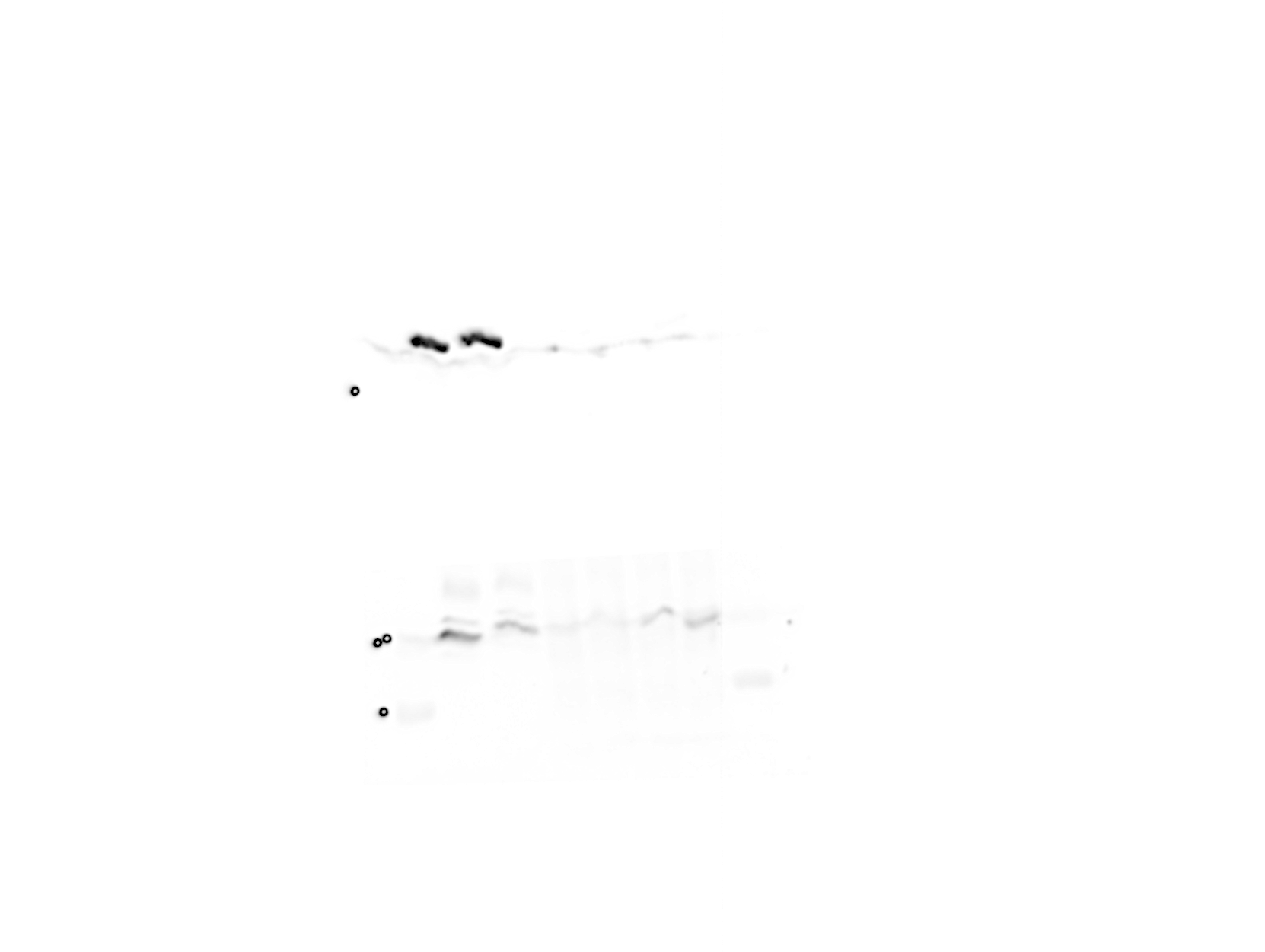

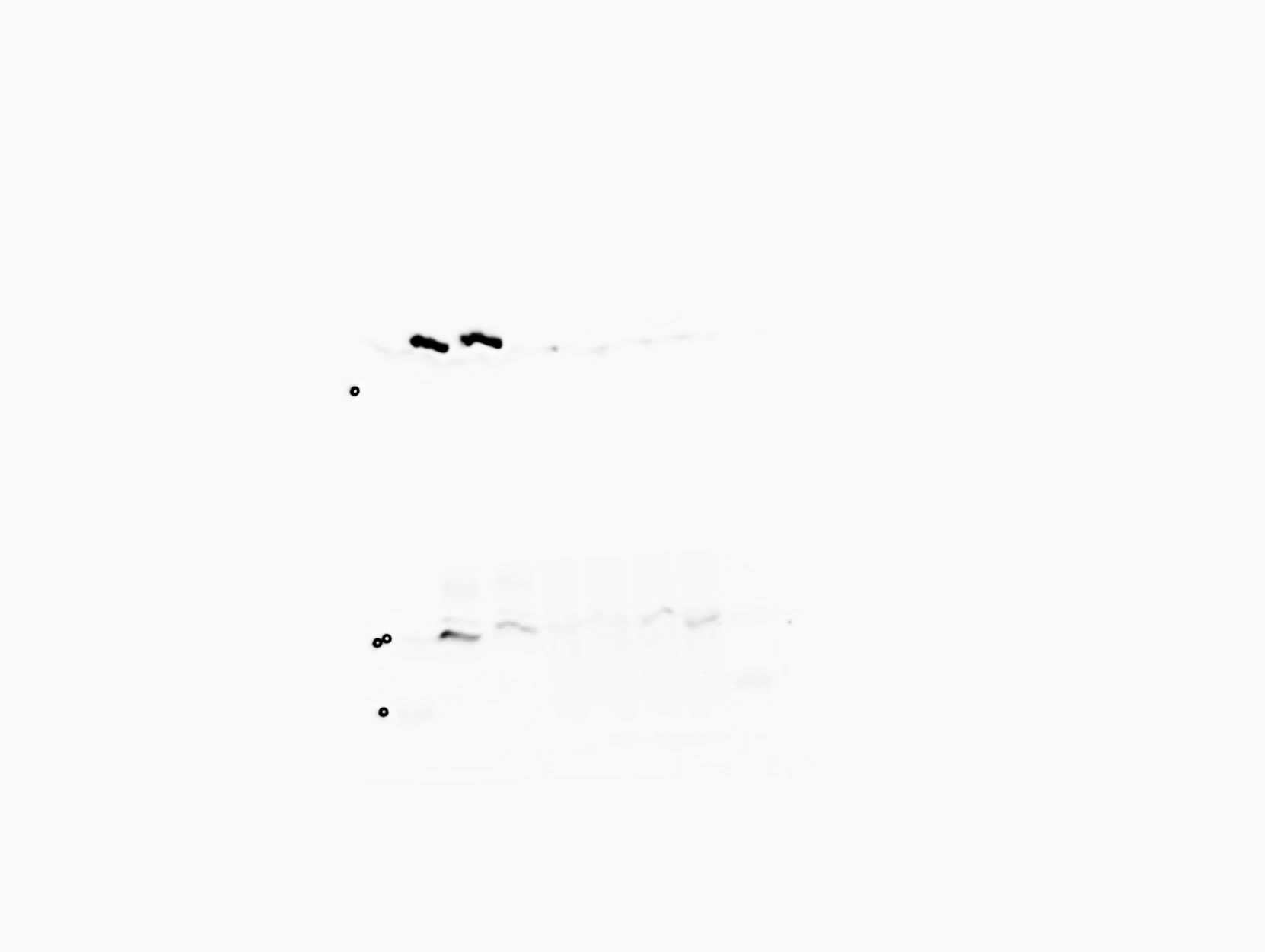

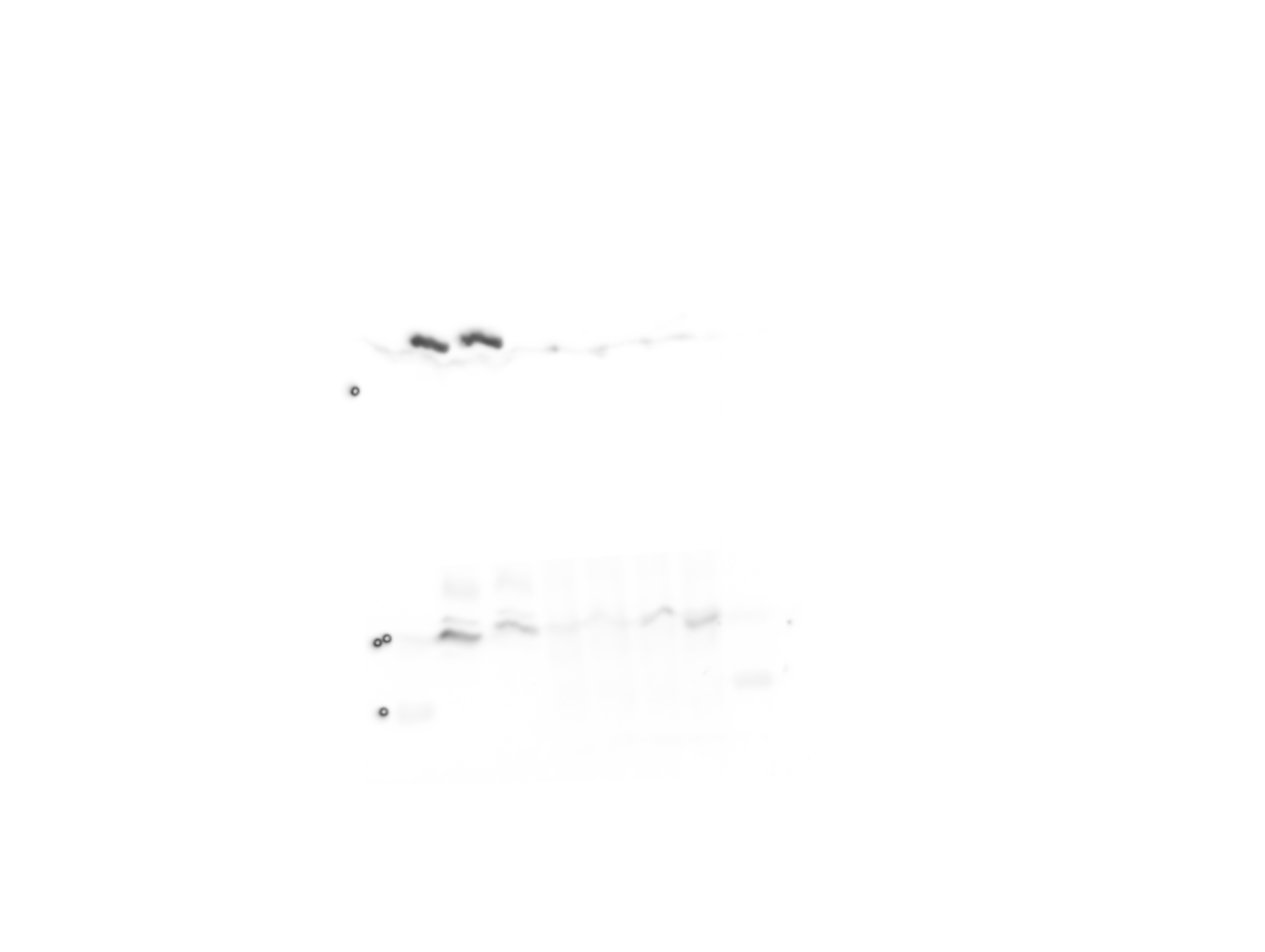


**β-actin**

**cFos**

**Sig-1R (5460)**

**Sham**

**SNI**

**Sham**

**SNI**

**Input**

**IP:Sig-1R**


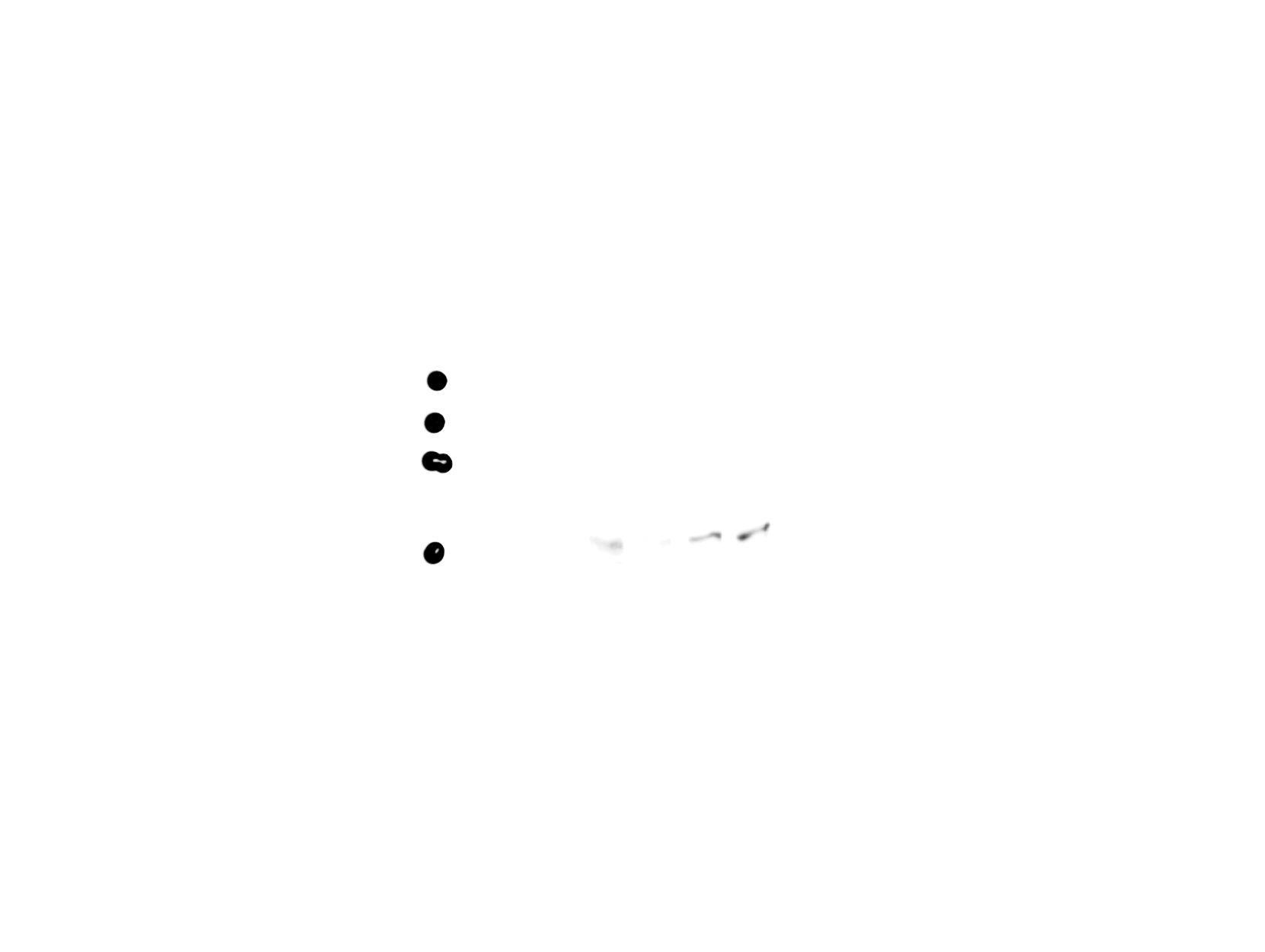


**cFos/Sig-1R ratio**

**1.00**

**1.51**

Fig. S7

**Figure S7. Sig-1R increases its interaction with transcription factor cFos after SNI.** Blot shows the Sig-1R antibody (sc-137075) pulling down cFos in DRGs from Sham or animals 14 days after SNI. The ratio of cFos to Sig-1R after IP increases after SNI (right panel).

(+)PTZ

(100μM)

**2 min**

**200nM**

K^+^

K^+^

K^+^

K^+^

Vehicle

2 mM Ca^2+^ bath

0 mM Ca^2+^ bath

**(A)**

**(B)**

Vehicle

(+)PTZ

(100μM)

**2 min**

**200nM**

K^+^

K^+^

K^+^

K^+^

Tyrode

Tyrode

Dantrolene

Dantrolene

Fig. S8

**Figure S8. The** **effect of sigma-1 receptor agonist (+)pentazocine on** **activity-induced Ca^2+^ transient in sham DRG dependents on Ca^2+^ entry through VGCCs but not calcium-induced calcium release (CICR). (A)** The effect of (+)pentazocine ((+)PTZ; 100 µM) in reducing K^+^ (50 mM, 3sec)-evoked intracellular calcium transient ([Ca^2+^]_c_) is dependent on extracellular Ca^2+^ (2 mM). Data representative of 5 or 8 repetitions respectively with no significant inter-assay variations because the ratio of peak inhibitions in each set of tests did not differ (left bar or right bar; right panel). **(B)** (+)PTZ does not apparently block CICR because the residual [Ca^2+^]_c_ can be blocked by ryanodine receptor blocker dantrolene. Representative traces demonstrate that ryanodine receptor blocker dantrolene (10 μM) depresses CICR portion of the depolarization-induced (50 mM K^+^, 3 sec; 2 mM Ca^2+^ bath solution) [Ca^2+^]_c_ in vehicle or (+)PTZ (100 μM)-treated control sensory neurons. Summary data (right panel) show no significant inter-assay variations in those tests (see explanation in (A)). Data represent means ± SEM; Student’s t-test.
